# Imbalances in TCA, Short Fatty Acids and One-Carbon Metabolisms as Important Features of Homeostatic Disruption Evidenced by a Multi-Omics Integrative Approach of LPS-Induced Chronic Inflammation in Male Wistar Rats

**DOI:** 10.3390/ijms23052563

**Published:** 2022-02-25

**Authors:** Julia Hernandez-Baixauli, Nerea Abasolo, Hector Palacios-Jordan, Elisabet Foguet-Romero, David Suñol, Mar Galofré, Antoni Caimari, Laura Baselga-Escudero, Josep M Del Bas, Miquel Mulero

**Affiliations:** 1 Eurecat, Centre Tecnològic de Catalunya, Unitat de Nutrició i Salut, 43204 Reus, Spain; julia.hernandez@eurecat.org (J.H.-B.); antoni.caimari@eurecat.org (A.C.); laura.baselga@eurecat.org (L.B.-E.); 2 Eurecat, Centre Tecnològic de Catalunya, Centre for Omic Sciences (COS), Joint Unit Universitat Rovira i Virgili-EURECAT, 43204 Reus, Spain; nerea.abasolo@eurecat.org (N.A.); hector.palacios@eurecat.org (H.P.-J.); elisabet.foguet@eurecat.org (E.F.-R.); 3 Eurecat, Centre Tecnològic de Catalunya, Digital Health, 08005 Barcelona, Spain; david.sunol@eurecat.org (D.S.); mar.galofre@eurecat.org (M.G.); 4 Department of Biochemistry and Biotechnology, Universitat Rovira i Virgili, 43007 Tarragona, Spain; 5 Nutrigenomics Research Group, Department of Biochemistry and Biotechnology, Universitat Rovira i Virgili, 43007 Tarragona, Spain

**Keywords:** chronic inflammation, lipopolysaccharide, biomarker, metabolome, microbiome, energy metabolism, one-carbon metabolism, mitochondria

## Abstract

Chronic inflammation is an important risk factor in a broad variety of physical and mental disorders leading to highly prevalent non-communicable diseases (NCDs). However, there is a need for a deeper understanding of this condition and its progression to the disease state. For this reason, it is important to define metabolic pathways and complementary biomarkers associated with homeostatic disruption in chronic inflammation. To achieve that, male Wistar rats were subjected to intraperitoneal and intermittent injections with saline solution or increasing lipopolysaccharide (LPS) concentrations (0.5, 5 and 7.5 mg/kg) thrice a week for 31 days. Biochemical and inflammatory parameters were measured at the end of the study. To assess the omics profile, GC-qTOF and UHPLC-qTOF were performed to evaluate plasma metabolome; ^1^H-NMR was used to evaluate urine metabolome; additionally, shotgun metagenomics sequencing was carried out to characterize the cecum microbiome. The chronicity of inflammation in the study was evaluated by the monitoring of monocyte chemoattractant protein-1 (MCP-1) during the different weeks of the experimental process. At the end of the study, together with the increased levels of MCP-1, levels of interleukin-6 (IL-6), tumour necrosis factor alpha (TNF-α) and prostaglandin E2 (PGE2) along with 8-isoprostanes (an indicative of oxidative stress) were significantly increased (*p*-value < 0.05). The leading features implicated in the current model were tricarboxylic acid (TCA) cycle intermediates (i.e., alpha-ketoglutarate, aconitic acid, malic acid, fumaric acid and succinic acid); lipids such as specific cholesterol esters (ChoEs), lysophospholipids (LPCs) and phosphatidylcholines (PCs); and glycine, as well as N, N-dimethylglycine, which are related to one-carbon (1C) metabolism. These metabolites point towards mitochondrial metabolism through TCA cycle, β-oxidation of fatty acids and 1C metabolism as interconnected pathways that could reveal the metabolic effects of chronic inflammation induced by LPS administration. These results provide deeper knowledge concerning the impact of chronic inflammation on the disruption of metabolic homeostasis.

## 1. Introduction

Inflammation is a part of a complex biological process characterized by the activation of immune and non-immune cells that protect the host from bacteria, viruses, toxins and infections, eliminating pathogens and promoting tissue repair and recovery [1]. Chronic inflammation is defined as a long-term inflammation lasting for prolonged periods, from several months to years in humans, and is characterized by the sustained elevation of inflammatory cytokines in serum due to the failure to resolve acute inflammation, oxidative stress or metabolic dysfunction [2]. Generally, the extent and effects of chronic inflammation vary depending on the cause of the injury and the ability of the body to repair and overcome the damage [3]. One of the most remarkable medical discoveries of the past two decades has been that the inflammatory processes are intimately involved in the onset of numerous NCDs such as cardiovascular diseases (CVDs), neurodegenerative processes, diabetes, cancer, auto-immune disease, non-alcoholic fatty liver disease (NAFLD) as the main liver disease and renal disease, among others [3]. Thus, chronic inflammation might be understood as a main risk factor, and, consequently, we have a long way to go before achieving full understanding about the role that chronic inflammation plays in disease risk, biological aging and NCDs evolution and mortality [4].

Current research on inflammation has focused on the causes of chronic inflammation, the discovery of inflammation-associated biomarkers and the associations between inflammation and disease. Nevertheless, further research is needed to better understand this condition and its progression towards the development of individual disease status [5]. In line with this, different studies have shown that current biomarkers of inflammation need complementary information to be applied for the monitoring of chronic inflammation. For example, monitoring the levels of general biomarkers of inflammation has been shown as a promising strategy for the prediction of morbidity and mortality in cross-sectional and longitudinal studies related to inflammation in aging [6]. However, alteration of cytokines (e.g., interleukin-1 (IL-1) and interleukin-6 (IL-6)) was discordant between studies [7,8,9]. Hereby, the assessment of chronic inflammation as a risk factor requires novel biomarkers and approaches to complement the information provided by the classical ones.

Consequently, there is an increasing demand for novel and growing sources of potentially promising biomarkers, such as adipokines, cytokines, metabolites and microRNAs, that are related to inflammation, as well as for a multi-dimensional approach/integration of them. This could bring huge improvements in the personalized prevention and treatment of some inflammatory-related pathologies [10]. In this regard, metabolomics is a very powerful tool for the study of the living organism thanks to the direct involvement of metabolite homeostasis in the final phenotype, which in turn is affected by the proper functioning of higher levels of biochemical organization, including the genome, the transcriptome and the proteome [11]. Recently, the field of integrative omics has been growing as an important tool for the prognosis and diagnosis of different diseases by investigating the endogenous levels of metabolites of different biofluids (i.e., plasma/serum and urine) [11,12].

Additionally, besides the need for a deeper understanding of chronic inflammation, there is a strong need to target accurate animal models that reflect the biochemical and metabolic manifestations of the homeostatic disruption, which is generated by chronic inflammation. In this sense, a variety of studies have developed models to mimic acute or chronic inflammation using chemical or biological stimuli [13,14]. However, the isolation of chronic inflammation is challenging because it appears concomitantly with other conditions, and it does not exist separately in humans. Experimentally, one of the preferred stimuli for inducing chronic inflammation is the administration of lipopolysaccharide (LPS), a structure found in the outer membrane of gram-negative bacteria, which could be injected either intravenously or intraperitoneally, with different doses and frequency [14,15,16,17]. Furthermore, adjustment of administration, dose and frequency, together with additional approaches, allowed the development of different models of chronic inflammation diseases (e.g., CVDs or NAFLD) [18,19]. Nevertheless, it has been reported that repeated administration of LPS may reduce the ability of animals to respond to endotoxin, due to the development of endotoxin tolerance and the subsequent decrease in the inflammatory response [20]. For this reason, different procedures have been explored to overcome animal-generated endotoxin resistance, considering time and cost. One of the cutting-edge approaches consists of using LPS infusion delivered by time-release pellets for at least 60 days [19,21], but this technique failed to induce chronic inflammation, suggesting that intermittent injection of LPS on different days might be more effective in rats [14,22]. Several studies with encouraging results appeared to solve the LPS-resistance problem by means of intraperitoneal (IP) injections thrice a week, starting with doses between 1 ng/kg and 20 mg/kg and steadily increasing LPS doses [14,20,23].

In the present work, we hypothesized that chronic inflammation is accompanied by a characteristic metabolic signature that might allow the detection of a different range of inflammatory states affecting metabolism homeostasis. When applied together with classic inflammatory biomarkers, this metabolic signature might provide valuable information on chronic inflammation as a risk factor for the development of metabolic alterations leading to different diseases such as CVDs, NAFLD and neurodegeneration, among others. Therefore, the objective was to assess new metabolomic features of chronic inflammation to gain a deeper understanding of the metabolic signatures associated with inflammatory-involved diseases. To this end, we stablished a model of chronic inflammation in rodent based on injections of LPS at increasing doses. Subsequently, we interrogated the chronic inflammation model to unravel the affected metabolic pathways and identify characteristic profiles employing a multi-omics approach, including the metabolome of different biofluids (i.e., plasma and urine). Finally, we propose a biomarker/metabolic profile for the assessment of metabolic alterations associated with chronic inflammatory states underlying different metabolic diseases. Furthermore, we highlight the corresponding metabolic pathways that might be most altered and therefore studied to understand the underlying mechanisms.

## 2. Results

### 2.1. Characterization of the LPS-Induced Inflammation Model

**Figure 1:**
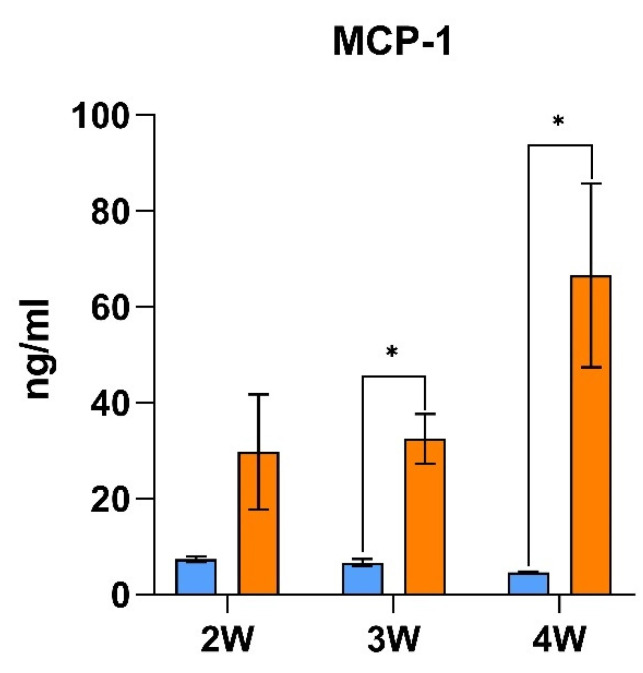
Assessment of inflammation levels during the experimental period by MCP-1 monitoring. The results are presented as the mean ± SEM (*n* = 10 animals per group). * indicates significant differences using *t*-student test between CON and LPS group (*p* < 0.05). Abbreviations: MCP-1, monocyte chemoattractant protein-1; W, study’s week.

The stability of inflammation was evaluated during the entire experimental period thanks to the monitorization of monocyte chemoattractant protein-1 (MCP-1) during the second week, the third week and at the end of the study (Figure 1). The results indicate that MCP-1 was elevated during the entire experimental procedure, indicating a constant effect of the LPS treatment in inflammation. Thus, the recurrent administration of LPS induced a stable inflammatory state. At the end of the study, the impact of LPS treatment on inflammation was evaluated by assessing classical inflammatory biomarkers in plasma, which showed an increase in MCP-1, as well as an increase in tumour necrosis factor alpha (TNF-α), interleukin-6 (IL-6) and prostaglandin E2 (PGE2) (Table 1). These results indicate an alteration in inflammation during the experimental period and at the end of the study due to the LPS treatment. As both conditions (inflammation and oxidative stress) are often present together, the level of oxidative stress was also assessed by measuring urinary 8-isoprostanes level, which was increased by a factor of 5 in the LPS-induced inflammation model. Therefore, an alteration of oxidative levels was also observed in the LPS group.

**Figure 2:**
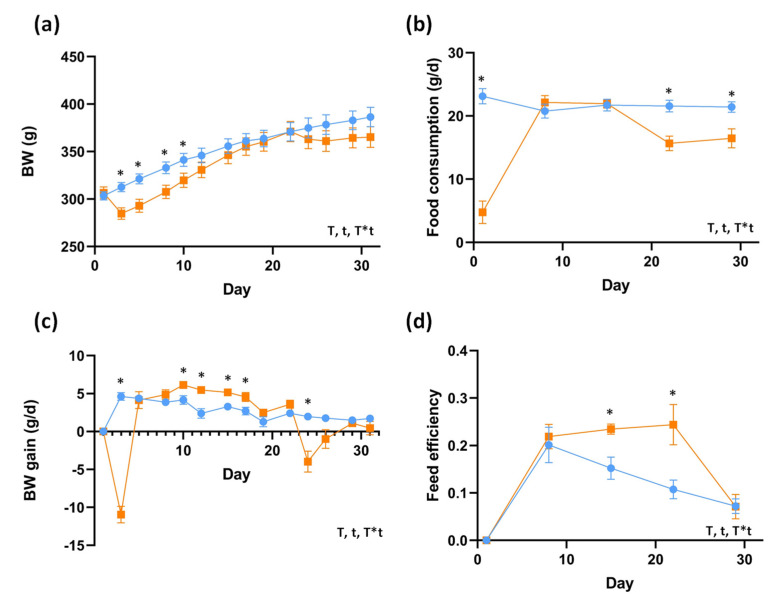
Body weight (BW) (**a**), BW gain (**b**), food consumption (**c**) and feed efficiency (**d**) of the LPS-induced inflammation model. Blue represents CON group and orange LPS group. Each point of represented data corresponds to the mean ± SEM (*n* = 10 per group). * indicates significant differences using repeated-measures ANOVA followed by *t*-student test between CON and LPS group (*p* < 0.05). T indicates treatment effect; t, indicates time effect; T*t interaction between treatment and time. Abbreviations: BW, body weight.

Regarding other important characteristics of animals, body weight (BW) and food consumption were modified by LPS inoculation; thus, the decrease in BW and food consumption appeared to be related with the initial injection of LPS (Figure 2). Despite BW being similar between control (CON) group and LPS group at the end of the study (Figure 2a, Table 1), BW was significantly influenced by the LPS. During the entire experimental period, food consumption remained significantly decreased despite the different tendencies (Figure 2b, Table 1). It could be observed that BW gain was significantly decreased during the first days until a moment where the LPS group presented higher BW gain than the CON group (Figure 2c) and a corresponding improvement in feed efficiency (Figure 2d). Additionally, muscle weight was significantly decreased, while liver weight was significantly increased in the LPS group (Table 1). Among the most interesting changes in the biochemical parameters, plasma triglycerides (TG) were decreased, while liver TGs were increased (Table 1).

### 2.2. Plasma Metabolome of the LPS-Induced Inflammation Model

The plasma metabolomic approach was based on a global multiplatform analysis including 128 metabolites (Table S1). This platform is able to discriminate between metabolites implicated in lipid metabolism as TGs, diacylglycerols (DGs), phosphatidylcholines (PCs), cholesterol esters (ChoEs), lysophospholipids (LPCs) and sphingomyelins (SMs); carbohydrate metabolism (mainly the tricarboxylic acid (TCA) cycle); and amino acid metabolism, among other interesting metabolites. The summary of analysis is shown in Table S1, including the univariate, multivariate and prediction analysis. After the Mann–Whitney (MW) test, 40 out of 128 metabolites were significantly different, and the subsequent Benjamini–Hochberg (BH) correction highlighted 24 out of 47 different metabolites (Table 2).

**Figure 3:**
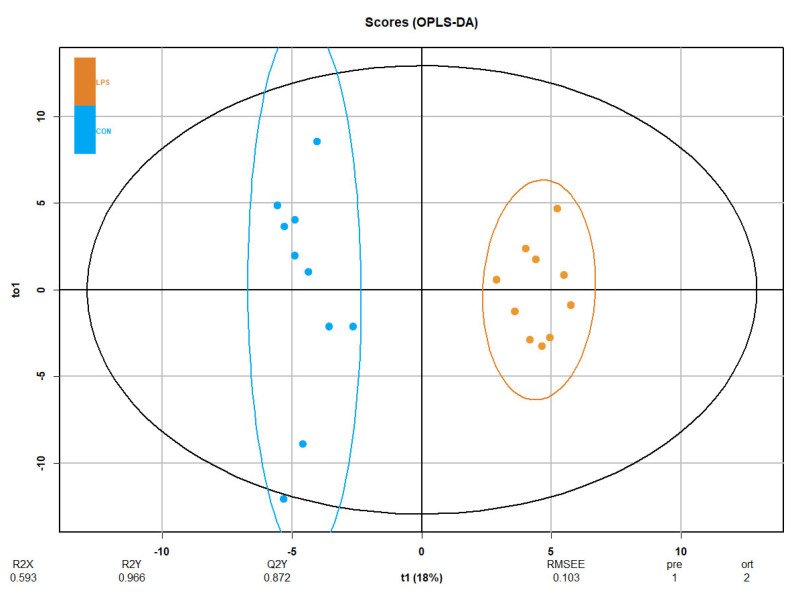
OPLS-DA of plasma metabolomics in the LPS-induced inflammation model. The Score plot is represented, and it includes the number of components and the cumulative R2X, R2Y and Q2Y. Blue represents CON group and orange LPS group (*n* = 10 animals per group).

Principal component analysis (PCA) was performed to explore and identify the largest source of variation in the data, showing a modest clustering (Figure S1). Additionally, orthogonal partial least-squares discriminant analysis (OPLS-DA), which performs classification tasks and could predict the class, showed clear differences between groups, confirming the robustness of the LPS-induced inflammation model (Figure 3). The proportion of variance in the plasma data explained by the model (R2X) was 59.34%. The percentage of Y variability explained by the model (R2Y) was 96.6%, and the estimation of the predictive performance of the models (Q2) was 87.2%, as it is greater than 50%; thus, the model is considered to have good predictability. The highest variable importance in projection (VIP) values is shown in Table 2, with alpha-ketoglutarate and malic acid being the most important discriminative metabolites (VIP > 2) in the model, followed by other metabolites (e.g., ChoE 22:6, fumaric acid and DG 36:4, among others). Finally, the feature importance was also assessed using RF focusing on the selected metabolites, thus, PC 34:0, malic acid, TG 54:7, alpha-ketoglutarate and succinic acid presented outstanding results in the evaluation of prediction power (Table S1).

### 2.3. Urine Metabolome of the LPS-Induced Inflammation Model

The urine metabolomic approach was based on untargeted ^1^H-NMR methodology detecting 33 metabolites related to metabolism of amino acids (e.g., phenylalanine, tyrosine and tryptophan metabolism; glycine, serine and threonine metabolism; alanine, aspartate and glutamate metabolism; glutathione metabolism; and taurine and hypotaurine metabolism) and energetic metabolism (e.g., TCA cycle, pyruvate metabolism, and glycolysis/gluconeogenesis) (Table S2). The summary of univariate and multivariate analysis is shown in Table S2. After the MW test, N,N-dimethylglycine was significantly altered in LPS versus the CON group. After the BH correction, none of these metabolites remained significantly modified. In the case of multivariant approaches, no clustering was distinguished in PCA (Figure S2), and OPLS-DA was not significative for predictive power (data not shown). Additionally, N,N-dimethylglycine, which is almost duplicated in the LPS group, is the metabolite presenting the highest RF value, as is shown in Table S2.

### 2.4. Microbiome of the LPS-Induced Inflammation Model

**Figure 4:**
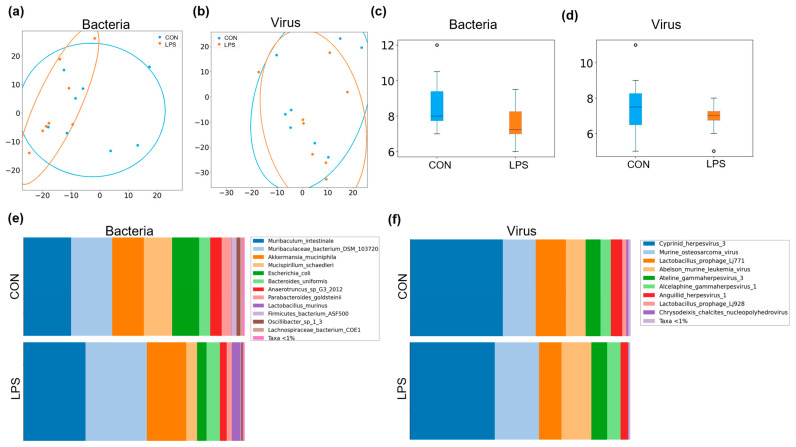
Summary of the microbiome statistical analysis in the LPS-induced inflammation model. Beta diversity: PCA plot calculated by Aitchison distance for bacteria (**a**) and virus (**b**). Alpha diversity (AU): chao1 index in bacteria (**c**) and virus (**d**). Taxonomic differences represented as relative distribution of species in bacteria (**e**) and virus (**f**); these figures show a bar graph at the level of both bacterial and viral species (relative %), comparing the animals in all groups. Blue represents CON group and orange LPS group (*n* = 8 animals per group).

In this research, the aim of the microbiota study was to enrich the full characterization of the effects of the LPS-induced inflammation model. The most abundant microbes were the bacterial ones, followed by virus and other microbes (less than 1%). For instance, 67% of the readings generated were assigned to bacteria and 33% to virus in CON group, and in the case of the LPS groups, the bacteria level was decreased to 58% and virus was increased to 42% in comparison to CON group. On the one hand, beta diversity, which is represented by a PCA constructed with the Aitchison distances, was not clearly clustering the different groups in bacteria (Figure 4a) and virus (Figure 4b). In the same way, PERMANOVA results were not statistically different, neither in bacteria (F = 1.11, *p*-value = 0.34) nor virus (F = 1.10, *p*-value = 0.30). On the other hand, alpha diversity, which is the measure of richness in the same group, was statistically decreased in bacteria (*p*-value < 0.01, Figure 4c) and virus (*p*-value < 0.01, Figure 4d) considering Chao1 index.

In terms of the bacterial microbiome, the communities of both groups were mostly formed by the phyla *Bacteroidetes* (CON: 49% and LPS: 64%), *Verrucomicrobia* (CON: 14% and LPS: 18%), *Firmicutes* (CON: 11% and LPS: 9%), *Deferribacteres* (CON: 13% and LPS: 5%), *Proteobacteria* (CON: 12% and LPS: 4%) and *Actinobacteria* (CON: 1% and LPS: 0%). Focusing on bacterial species (Figure 4e), 19 species were found with a relative abundance above 0.01% (Table S3), and 3 of them were statistically different after the MW test: Muribaculum intestinale (*p*-value = 0.03, *q*-value = 0.55, CON: 21% and LPS: 28%) and *Lachnospiraceae bacterium A4* (*p*-value = 0.03, *q*-value = 0.55, CON: 0.13% and LPS: 0.32%) were increased, while *Firmicutes bacterium ASF500* was decreased (*p*-value = 0.03, *q*-value = 0.61, CON: 2.27% and LPS: 0.36%). In terms of the virus microbiome, 14 species were found with a relative abundance above 0.01% (Table S4), and 2 species were statistically increased after the MW test in LPS group, an unknown virus of *Alphabaculovirus genera* (*p*-value = 0.02, *q*-value = 0.38, CON: 0.1% and LPS: 0.27%) and an unknown virus of *Pestivirus* genera (*p*-value = 0.03, *q*-value = 0.52, CON: <0.01% and LPS: 0.23%).

### 2.5. Multi-Omics Data Integration

The multi-omics integrative analysis was performed with the Data Integration Analysis for Biomarker discovery using Latent cOmponents (DIABLO) method that was able to discern a multi-omic profile of eight plasma metabolites (alpha-ketoglutarate, PC 34:0, aconitic acid, LPC 16:0 e, malic acid, SM 42:3, PC 38:4, ChoE 18:3), six urine metabolites (N,N-dimethylglycine, fucose, citrate, dimethylsulfone, formate, 2-oxoglutarate) and five microbes (*Escherichia coli*, *Pestivirus Giraffe 1*, *Anaerotruncus sp G3 2012*, *Oscillibacter sp 1 3*, *Firmicutes bacterium ASF500*). The correlation method, which is built on the Generalised Canonical Correlation Analysis (GCCA), revealed a correlation between the three data sets with coefficients above 0.6: plasma and urine metabolome (r = 0.79); plasma metabolome and microbiome (r = 0.64); and urine metabolome and microbiome (r = 0.67). The three data sets were able to discriminate between groups (Figure S3); in this case, plasma features presented major impact in the correlation between data sets (Figure S4). The variable effect in the first component, which explains the highest correlations between data, and the impact of each feature on the data sets, are shown in Figure S5a,c,e for plasma metabolomics, urine metabolomics and metagenomics, respectively.

**Figure 5:**
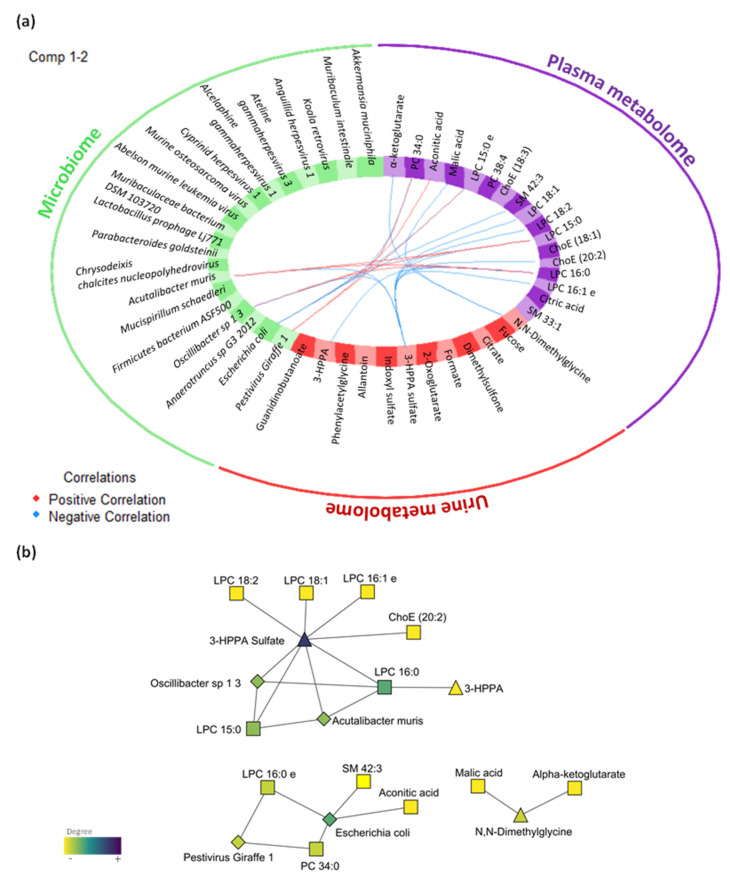
Multi-omics integration of plasma metabolome, urine metabolome and microbiome in the LPS-induced inflammation model. (**a**) Circos plot output from DIABLO. Each quadrant indicates the type of features: plasma metabolites (purple), urine metabolites (red), microbiome (green). (**b**) Further visualization of the network from DIABLO using Cytoscape. The shape of the features indicates the type of feature: plasma metabolites (square), urine metabolites (triangle) and metagenomics (diamond). The colour indicates the degree of each feature in the network (i.e., nodes with more connections). Abbreviations: ChoE, cholesterol ester; TG, triglyceride; PC, phosphatidylcholine; SM, sphingomyelin; LPC, lysophospholipid.

The correlation between the features is represented in Figure 5, to show connections within and between blocks and expression levels of each variable according to each class. In the DIABLO model, the 3-HPPA sulphate (urine metabolite) was negatively correlated with several LPCs and other plasma metabolites together with some bacteria (i.e., *Oscillibacter sp 1 3* and *Escherichia coli*). Another urine metabolite, N,N-dimethylglycine, was correlated with alpha-ketoglutarate and malic acid, which are metabolites identified in plasma related to the TCA cycle. In this sense, the general correlation between plasma and urine metabolites was negative, while the correlation between microbes and plasma metabolites was positive.

Finally, the overall error rate was calculated (0.3) for the first component to evaluate the performance of the omic profile generated by DIABLO. To give an idea, receiver operating characteristic (ROC) curve analysis showed that the optimal omic profile with the combination of eight plasma metabolites effectively separated both groups with an area under the curve (AUC) of 1 (*p*-value < 0.01, Figure S5b). A combination of six plasma metabolites optimally dichotomized the groups with an AUC of 0.89 (*p*-value < 0.01, Figure S5d). In the microbiome, the microbes presented an AUC of 0.95 (*p*-value < 0.01, Figure S5f). These results support these features as key mediators of the LPS-induced inflammation model considering plasma metabolome, urine metabolome and microbiome. In this sense, the best correlations were associated with plasma metabolome, as was elucidated in the previous statistical analysis. Thus, the most optimal source of biomarkers in this study was the plasma.

## 3. Discussion

The evolution of chronic inflammation causes “silent” damage in the development of NCDs, partly favoured by the lack of knowledge about its mechanism and evolution. In this sense, the current study presents deeper understanding of this condition and novel insights through the use of a rodent inflammatory model induced by intermittent (1- and 2-day intervals) and increasing LPS IP injections (0.5, 5 and 7. 5 mg/kg). Hence, this approach was tried to overcome the problems related to LPS-habituation and ineffective establishment of chronic inflammation in rodent models. In previous studies, doses of 2 mg/kg reduced the survival rate to 50% compared to control groups [14], while in our case, the survival rate was 100% compared to the untreated animals. In other studies comparing the LPS in humans and rodents, LPS injections of 10 mg/kg in rodents showed similar scalable levels in humans [16], which are comparable to LPS levels in human diseases [24]. Consequently, we suggest that the rodent ability to tolerate frequent LPS challenges facilitates the dose increment up to 7.5 mg/kg, used in our approach, to carry out chronic studies related to inflammation. Furthermore, the LPS reached levels that are close to previously described LPS levels related to human diseases [20]. The intermittent and increasing LPS treatment, which resulted in BW changes due to the effect of LPS on appetite, was reflected in food consumption, which was in agreement with other studies [14,25]. The initial injection had a huge impact on BW and food consumption; then, the animals compensated for the initial response with higher feed efficiency rates, and at the end of the study, both groups presented the same pattern. These changes are not in line with the general features of NCDs, as in this chronic inflammation model, the intention was to isolate the risk factor, considering that it would not occur in a real individual.

The mechanism of inflammation induced by LPS consists of the activation of Toll-like receptors (TLRs), which lead to the activation of macrophages and lymphocytes and the production of inflammatory cytokines (i.e., TNF-α, IL-6, PGE2) [26], which is in agreement with our experimental results. Although those cytokines have several roles in different tissues and cell types related to the inflammatory response, many cytokines have other effects on neuroendocrine and metabolic functions and on the maintenance of tissue homeostasis in general [27]. In the case of MCP-1, which attracts inflammatory monocytes and stimulates the production of other cytokines, it was the selected cytokine to monitor the sustained inflammation during the experimental procedure [28]. Those inflammatory monocytes have been involved in low-grade inflammation and altered lipid metabolism through the release of various pro-inflammatory mediators [29]. The systemic increase in oxidative stress detected in the LPS group in urine is associated with the immune response that could be attempting to kill the invading agents through the releasing of toxic content from cells (including reactive oxygen species (ROS)) [30].

**Figure 6:**
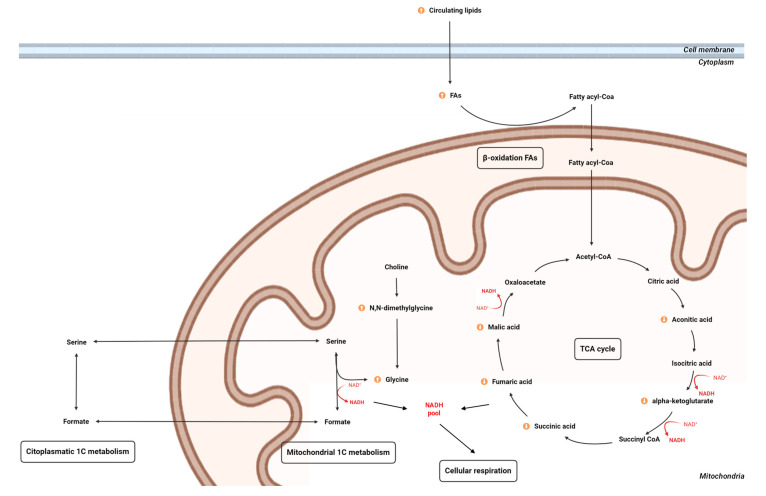
A schematic overview of the metabolic pathways involved in the LPS-induced inflammation model. In the context of mitochondria, TCA cycle is the nexus between FAs through β-oxidation disruption, glycine through 1C metabolism and cellular respiration. Abbreviations: FA, fatty acid; NAD+/NADH, nicotinamide adenine dinucleotide oxidized and reduced.

As far as we know, this study is the first to evaluate the omic profile of a chronic inflammation model with the objective of elucidating the metabolic mechanism and finding potential biomarkers for early detection of metabolic alterations associated with chronic inflammation, which leads to the onset of different diseases. The study of chronic systemic inflammation is important because it can trigger and propagate metabolic inflexibility; this fact can cause a vicious circle, because such metabolic inflexibility can also trigger systemic inflammation [31]. Recent studies focused on those pathologies (CVDs [32], NAFLD [33], arthritis [34], obesity and diabetes [35]) have suggested an alteration in metabolomic and lipidomic signatures implicated in the onset of NCDs development. Nevertheless, these signatures cannot discern between chronic inflammation and other factors influencing the development of the disease. In our approach, we have detected three key pillars of metabolic disruption associated with chronic inflammation: lipidic metabolism (mainly focused on specific fatty acids (FAs) metabolism), TCA cycle and one-carbon (1C) metabolism, as summarized in Figure 6.

Lipids play an important role in the pro-inflammatory and anti-inflammatory response; thus, lipidic dynamics disruption leads to unbalanced homeostasis and the subsequent development of pathologies. In fact, lipids act as mediators on important immune receptors to induce chronic tissue inflammation that leads to adipocyte and metabolic dysfunction [36]. In our LPS-induced inflammation model, diverse types of lipids were increased. ChoEs, PCs and LPCs stand out among other lipids as the important ones, due to their influence in chronic inflammation and interactions in energetic metabolism.

Identification and quantification of circulating FAs, which are involved in critical cellular functions such as storage of energy and signalling pathways, have attracted attention as potential inflammatory status biomarkers [37]. The leading circulating FAs are made up of even-chains of 18 to 22 carbons, and regarding the saturations, we could find poly-unsaturated FAs (PUFAs) and saturated FAs (SFAs) on the LPS-induced chronic inflammation rodent model. Accumulating evidence suggests that ChoE 18:0, which is the main even SFA altered in our study, is the main even-chain SFA associated with lipid metabolism, liver function, glycaemic control and chronic inflammation leading to CVDs [38]. Additionally, ChoE (18:0) accumulation in macrophages has been related to the induction of inflammation by TLR 4/2 inducing endoplasmic reticulum stress-mediated apoptosis [39] and is considered to induce lipotoxicity in adipocytes [40]. These hypertrophied adipocytes enriched in SFAs secrete pro-inflammatory agents that promote systemic inflammation [41] and the subsequent obesity-associated adipose tissue inflammation [42].

Circulating phospholipids (LPCs and PCs) have been reported to reflect FAs metabolism, thus pointing to long-term storage of FAs indicating a long-standing exposure to FAs that are classically used as dietary biomarkers [43,44]. On the one hand, in the current experimental approach, circulating LPCs, which were composed by SFAs of 16 and 18 carbons, were also affected. Interestingly, phospholipids have been associated with pro-inflammatory and pro-atherogenic activities, which are critical factors underlying CVDs and several pathological conditions, in consistence with previous studies [38,45,46,47]. In fact, LPC can induce expression of COX2, a key pro-inflammatory mediator, via the p38/CREB or ATF-1 pathways in vascular endothelial cells [48]. For example, a study focused on asthma, which is a chronic inflammatory disease of the airways, presented elevated LPC 16:0 and LPC 18:0 together with activity increase in phospholipase A_2_ (PLA_2_) [49]. On the other hand, PCs, which carry two chains of FA, presented a predominance of chains from 16 to 20 carbons, with the SFAs predominant over MUFAs and PUFAs. This could be indicative that chronic inflammation is characterized by an increase in even SFAs between 16 and 18 carbons [50,51,52]. This observation suggests that chronic inflammation could induce long-term changes in the metabolism of SFAs through increasing their circulation bound to phospholipids [53]. Similarly, previous studies have shown that increased levels of PCs are positively correlated with obesity, insulin resistance, tumours and psoriasis among other inflammatory pathologies [54].

Therefore, after activation of cytosolic PLA_2_, important lipid mediators of inflammatory response (FAs and LPCs) are generated, among which PUFAs are the most interesting hydrolysed FAs regarding their effects during inflammation [55]. For instance, ChoE 20:4, commonly known as arachidonic acid, is metabolized to form eicosanoids by the action of cyclooxygenases (COX1 and COX2), which generates prostaglandins and thromboxanes, or by lipoxygenases, that subsequently generate leukotrienes and lipoxins, which are associated with a pro-inflammatory response [26]. Alternatively, ChoE 18:3, commonly known as linolenic acid, is a precursor of ChoE 22:6 or docosahexaenoic acid or omega 3, and it is associated with anti-inflammatory response. The synthesis of ChoE 20:4 from ChoE 18:2 seems to be reduced in favour of the synthesis of ChoE 22:6 from ChoE 18:3 due to both syntheses competing for the same enzymes (Δ6 desaturase, elongase and Δ5 desaturase) [56]. The significant increase in ChoE 18:3 (precursor of ChoE 22:6) instead of ChoE 18:2 (precursor of ChoE 20:4) suggests that the synthesis of ChoE 22:6 reaction is favoured.

During periods of stress in the cell, intermediary metabolites of the TCA cycle, which occurs completely in mitochondria, can be released, acting as a danger signals in cytosol and regulating immune response [57]. The decrease in TCA intermediaries (i.e., alpha-ketoglutarate, aconitic acid, malic acid, fumaric acid and succinic acid) might be indicative of a systemic inhibition of the intracellular TCA cycle (Figure 6) that could be associated with the regulation of immune response and activation of other energy pathways [58]. In fact, metabolic flexibility is essential for immune function; during immune response, the immune cells shift to aerobic glycolysis for energy production, a less-efficient but fast-acting pathway [59]. Additionally, the switch to glycolysis enables glycolysis, and TCA cycle intermediates may be used as key sources of carbon molecules for biosynthesis of nucleotides, amino acids and lipids [59]. Several metabolomic studies point out the decrease in TCA cycle activity as a key characteristic of chronic inflammation, related to alterations in lipid and fatty acid metabolism [60,61,62]. The connection between TCA cycle and FAs could be due to the β-oxidation of FAs. In fact, FAs enter the cell to be degraded into acetyl-CoA in the mitochondria as the end product of β-oxidation; this end product is the starting point of the TCA cycle [63].

Furthermore, other metabolites could be related to the inhibition of TCA cycle activity as 1C-metabolism-related metabolites (Figure 6). Specifically, 1C metabolism consists in methionine and folate cycles, involving multiple molecules of the cytosolic and mitochondrial compartments; moreover, the folate cycle is a major player in NADPH generation. In fact, production of NADH by mitochondrial 5,10-methylene-tetrahydrofolate (THF) dehydrogenase activity links 1C metabolism to the respiratory state of the cell. Glycine (circulating in plasma) and N,N-dimethylglycine (excreted in urine), which were increased in the metabolome, are amino acids essentially involved in the modulation of oxidative stress presenting antioxidant activity. For instance, previous experimental evidence has been generated in favour of the anti-inflammatory, immunomodulatory and cytoprotective effects of glycine [64]. Regarding the secreted urine amino acids, N,N-dimethylglycine, a glycine tertiary amino acid produced by the degradation of choline, was almost duplicated in the LPS group; this fact is in agreement with other animal studies suggesting that N,N-dimethylglycine enhances immune response due to intensifying oxygen utilization by tissue and complex with free radicals, as is the case for glycine [65]. Despite the antioxidant activity of glycine, their increased metabolism related to 1C metabolism has been associated with the development of tumorigenesis [66]. Maynard and Kanarek recently discovered the association of 1C cycle and TCA cycle through the accumulation of NADH in the mitochondria [67]. The 1C cycle becomes a major source of glycine and NADH when cellular respiration is inhibited, and the accumulated NADH inhibits the TCA cycle and slows proliferation due to its toxicity in high concentrations; thus, the unbalanced concentrations of NADH condition health and the pathological state (Figure 6). Additionally, mitochondrial NAD+/NADH ratios are maintained by oxidative phosphorylation, and initial experiments in isolated mitochondria showed that formate production from serine was respiration-dependent [68].This fact could be likely suggested in the present work linked to the increase in excreted formate in urine (despite being statistically significant). Within the mitochondria, 1C units can be made from serine, glycine, sarcosine or N,N-dimethylglycine and secreted into the cytosol as formate [69]. In this regard, the elevation in urinary excretion of formate was observed in folate-deficient rats [70]. Thus, formate may play a role in some of the pathologies associated with defective 1C metabolism, but there is a need for more studies to confirm this implication [71].

Interestingly, the profile obtained by DIABLO, in trying to maximize the correlation between the different data sets, highlights features from different data sets, although no differences were found in statistical analysis in urine metabolome and microbiome. This omic profile contemplated features that could complete the metabolic context of chronic inflammation. One of the most interesting relations is the association of N,N-dimethylglycine urine levels and TCA cycle intermediates (i.e., malic acid and alpha-ketoglutarate), which is in line with the previous discussion about the relation of 1C cycle and TCA cycle [67].

Although no major differences were found in the LPS- induced inflammation model, the microbiome has a close connection to systemic metabolism, highlighting the underlying crosstalk between the microbiome and inflammation [72]. However, the presence of these microbes or their metabolites may not represent the best source for biomarkers of inflammation, because the microbiome highly depends on the individual and the environment. Nevertheless, the microbiome can provide supplementary information indirectly related to the metabolome. In previous studies, low-grade inflammation has been related to alterations in the gut microbiota composition and increased plasma LPS levels [73]. In our case, as LPS has been administered, it is not originated by the individual’s microbiota, so the effect on inflammation due to the altered microbiota may be masked. For example, *Akkermansia muciniphila* has been shown to improve metabolic profiles by reducing chronic low-grade inflammation induced by chow diet in mice, linking this bacterium to the host immune system [74].

Finally, several shortcomings need to be taken into account in order to contextualize this experimental approach. The present study design should be considered as a pilot study due to the following factors: (1) there are limitations in obtaining a pure model of low-grade chronic inflammation, because although an attempt has been made to make the present model as close as possible to chronic inflammation, it is ultimately based on repeated induction of acute inflammation stimuli; (2) the low number of animals is not high enough (*n* = 10 per group) to provide a robust conclusion on biomarkers; (3) other omics approaches could be added to obtain a more robust metabolic profile; (4) urine is a complex fluid to collect in animals, as it is not possible to stablish the collecting time, which can lead to high variability of results. These issues should be considered for further experiments.

## 4. Materials and Methods

### 4.1. LPS-Induced Chronic Inflammation Model

Twenty 8-week-old male Wistar rats (Harlan Laboratories, Barcelona, Spain) were housed individually in a fully controlled environment including temperature (22 ± 2 °C), humidity (55 ± 5%) and light (12 h-light-dark cycle and lights on at 9:00 a.m.). The Animal Ethics Committee of the University Rovira i Virgili (Tarragona, Spain) approved all the procedures (code 10049). The experimental protocol followed the “Principles of Laboratory Care” and was carried out in accordance with the European Communities Council Directive (86/609/EEC).

**Figure 7:**
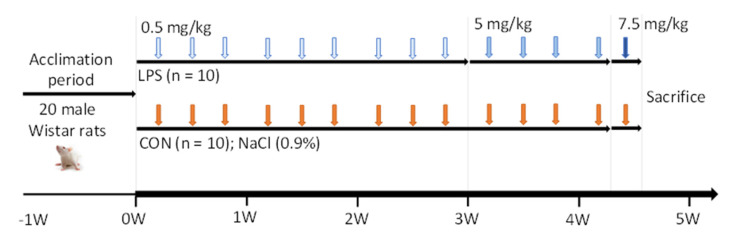
Schematic representation of the LPS-induced chronic inflammation model. The experimental model consisted of two groups that received intraperitoneal (IP) injections of increasing LPS and saline solution (NaCl 0.9%) for 31 days. Abbreviations: W, week; CON, control group; LPS, LPS-induced inflammation group.

After an acclimation period, animals were randomly assigned to two different groups considering similar average BW divided into two experimental groups (*n* = 10 animals per group): CON group and LPS-induced inflammation group. Regarding LPS (Sigma-Aldrich, St. Louis, MO, USA), a stock solution of 500 µg/mL was prepared in sterile saline solution (NaCl 0.9%) and stored in aliquots of 1 mL at −20 °C until administration. The experiment was conducted during the light phase (8:30–10:00 am) and was carried out for 31 days. In that period, rats were administered with increasing doses of LPS (LPS group) or saline solution (CON group) (Figure 7). The LPS group received nine IP injections of 0.5 mg/kg of LPS, four injections of 5 mg/kg and a final injection of 7. 5 mg/kg (6 h before the sacrifice).

To evaluate the effect of the LPS injections, mean daily BW gains were calculated for each group at each interval of three days per week and for the overall experimental period. Animals were allowed ad libitum access to food throughout the entire study period. Food consumption was measured once a week on days 1 (prior to dosing), 8, 15, 22, and 29, coinciding with the days of BW measurements. Mean food consumption was calculated for each group during each interval. Feed efficiency was also calculated for each group based on BW gain and food consumption data with the following equation: feed efficiency = food consumption (g/d)/BW gain (g/d).

### 4.2. Sample Collection

Blood was collected from the lateral saphenous vein in the second, third and fourth week to monitor the inflammation level. Urine was collected the day before the sacrifice following the hydrophobic sand method, which is less stressful for the animals [75]. For each rat, 300 g of hydrophobic sand was spread (LabSand, Coastline Global, Palo Alto, CA, USA) on the bottom of a mouse plastic micro-isolation cage. Urine was collected and stored with sodium azide (Sigma, St. Louis, MO, USA) as preservative every half an hour for 6 h and was finally pooled at the end of the session. On the day of the sacrifice, animals were euthanized by guillotine under anaesthesia (pentobarbital sodium, 50 mg/kg per BW) after 7 h of fasting. Blood was collected and centrifuged at 3000× *g* at 4 °C for 15 min to recover plasma. Tissues were rapidly removed, weighted and snap-frozen in liquid nitrogen (i.e., retroperitoneal white adipose tissue (RWAT), mesenteric white adipose tissue (MWAT), muscle, liver, and cecum). All the samples were stored at −80 °C until further analysis.

### 4.3. Plasma, Urine, and Liver Measurements

Enzymatic colorimetric kits were used for the determination of plasma total cholesterol (TC), TG, glucose (QCA, Barcelona, Spain) and non-esterified free fatty acids (NEFAs; WAKO, Neuss, Germany). Plasma concentrations of rat IL-6 (Cusabio Biotech Co., Wuhan, Hubei, China), MCP-1 (Thermo Fisher Scientific, Dublin, Ireland), TNF-α (Invitrogen, Vienna, Austria) and PGE2 (Bio-Techne Ltd., Minneapolis, MN, USA) were measured by enzyme-linked immunosorbent assay (ELISA) kits according to the manufacturer’s instructions. Additionally, urine 8-isoprostane was evaluated using an ELISA kit (Cayman chemical, Ann Arbor, MI, USA).

Liver lipids were extracted and quantified from a tissue piece of approximately 100 mg from the frozen liver using methods described previously [76]. Briefly, lipids were extracted with 1 mL of hexane:isopropanol (3:2, *v*/*v*), degassed with gas nitrogen before being left overnight under orbital agitation at room temperature protected from light. After an extraction with 0.3 mL of Na2SO4 (0.47 M), the lipid phase was dried with gas nitrogen and total lipids quantified gravimetrically before emulsifying as described previously [77]. TG, TC and phospholipids were assayed with commercial enzymatic kits (QCA, Barcelona, Spain).

### 4.4. Plasma Metabolome (GC-qTOF and UHPLC-qTOF)

Plasma metabolites were analysed by gas chromatography coupled with quadrupole time-of-flight (GC-qTOF). For the extraction, a protein precipitation extraction was performed by adding eight volumes of methanol:water (8:2, *v*/*v*) containing internal standard mixture (succinic acid-d4, myristic acid-d27, glicerol-13C3 and D-glucose-13C6) to plasma samples. Then, the samples were mixed and incubated at 4 °C for 10 min and centrifuged at 21,420× *g*, and the supernatant was evaporated to dryness before compound derivatization (metoximation and silylation). The derivatized compounds were analysed by GC-qTOF (model 7200 of Agilent, Santa Clara, CA, USA). The chromatographic separation was based on the Fiehn Method [78], using a J&W Scientific HP5-MS (30 m × 0.25 mm i.d.), 0.25 µm film capillary column and helium as carrier gas using an oven program from 60 °C to 325 °C. Ionization was done by electronic impact (EI), with electron energy of 70 eV and operated in full scan mode. The identification of metabolites was performed by matching two different parameters to a metabolomic Fiehn library (Agilent, Santa Clara, CA, USA): EI mass spectrum was considered stable and reproducible and as having good retention time. To avoid annotation errors, metabolites with very high molecular weights were cleared. After putative identification of metabolites, these were semi-quantified in terms of internal standard response ratio.

Plasma lipids were analysed by ultra-high-perfomance liquid chromatography coupled with quadrupole time-of-flight (UHPLC-qTOF). For the extraction of the hydrophobic lipids, a liquid–liquid extraction based on the Folch procedure [79] was performed by adding four volumes of chloroform:methanol (2:1, *v*/*v*) containing internal standard mixture (Lipidomic SPLASH^®^, Avanti Polar Lipids, Inc., Alabaster, AL, USA) to plasma. Then, the samples were mixed and incubated at −20 °C for 30 min. Afterwards, water with NaCl (0.8%) was added, and the mixture was centrifuged at 21,420× *g*. Lower phase was recovered, evaporated to dryness, reconstituted with methanol:methyl-tert-butyl ether (9:1, *v*/*v*) and analysed by UHPLC-qTOF (model 6550 of Agilent, Santa Clara, CA, USA) in positive electrospray ionization mode. The chromatographic consists in an elution with a ternary mobile phase containing water, methanol, and 2-propanol with 10 mM ammonium formate and 0.1% formic acid. The stationary phase was a C18 column (Kinetex EVO C18 Column, 2.6 µm, 2.1 mm × 100 mm) that allowed the sequential elution of the more hydrophobic lipids such as TGs, DGs, PCs, ChoEs, LPCs and SMs, among others. The identification of lipid species was performed by matching their accurate mass and tandem mass spectrum, when available, to Metlin-PCDL from Agilent containing more than 40,000 metabolites and lipids. In addition, chromatographic behaviour of pure standards for each family and bibliographic information was used to ensure their putative identification. After putative identification of lipids, these were semi-quantified in terms of internal standard response ratio using one internal standard for each lipid family.

A pooled matrix of samples was generated by taking a small volume of each experimental sample to serve as a technical replicate throughout the data set. As the study took multiple days, a data normalization step was performed to correct variation resulting from instrument inter-day tuning differences. Essentially, each compound was corrected in run-day blocks through quality controls, normalizing each data point proportionately.

### 4.5. Urine Metabolome (^1^H-NMR)

Urine metabolites were analysed by proton nuclear magnetic resonance (^1^H-NMR). The urine sample was mixed (1:1, *v*/*v*) with phosphate buffered saline containing with 3-(Trimethylsilyl)propionic-2,2,3,3-d4 acid sodium salt (TSP) (Sigma Aldrich, St. Louis, MO, USA) and placed on a 5 nm NMR tube for direct analysis by ^1^H-NMR. ^1^H-NMR spectra were recorded at 300 K on an Avance III 600 spectrometer (Bruker^®^, Bremen, Germany) operating at a proton frequency of 600.20 MHz using a 5 mm PBBO gradient probe. Diluted urine aqueous samples were measured and recorded in procno 11 using a one-dimensional ^1^H pulse. Experiments were carried out using the nuclear Overhauser effect spectroscopy (NOESY). NOESY presaturation sequence (RD–90°–t1–90°–tm–90° ACQ) suppressed the residual water peak, and the mixing time was set at 100 ms. Solvent presaturation with irradiation power of 150 μW was applied during recycling delay (RD = 5 s) and mixing time (noesypr1d pulse program in Bruker^®^, Bremen, Germany) to eliminate the residual water. The 90° pulse length was calibrated for each sample and varied from 11.21 to 11.38 ms. The spectral width was 9.6 kHz (16 ppm), and a total of 128 transients were collected into 64 k data points for each ^1^H spectrum. The exponential line broadening applied before Fourier transformation was of 0.3 Hz. The frequency domain spectra were manually phased and baseline-corrected using TopSpin software (version 3.2, Bruker, Bremen, Germany). Data were normalized in two different ways: probabilistically, to avoid differences between samples due to different urine concentrations, and by ERETIC. The acquired ^1^H-NMR were compared to references of pure compounds from the metabolic profiling AMIX spectra database (Bruker^®^, Bremen, Germany), HMDB and Chenomx databases for metabolite identification. In addition, we assigned metabolites by ^1^H-^1^H homonuclear correlation (COSY and TOCSY) and ^1^H-^13^C heteronuclear (HSQC) 2D NMR experiments and by correlation with pure compounds run in-house. After pre-processing, specific ^1^H-NMR regions identified in the spectra were integrated using MATLAB scripts run in-house. Curated identified regions across the spectra were exported to Excel spreadsheet to evaluate robustness of the different ^1^H-NMR signals and to give relative concentrations.

### 4.6. Microbiome Analysis (Shotgun Metagenomics Sequencing)

DNA was extracted from faeces using the PowerSoil DNA extraction kit (MO BIO Laboratories, Carlsbad, CA, USA) following the manufacturer’s protocol. Between 400 and 500 ng of total DNA was used for library preparation for Illumina sequencing employing Illumina DNA Prep kit (Illumina, San Diego, CA, USA). All libraries were assessed using a TapeStation High Sensitivity DNA kit (Agilent Technologies, Santa Clara, CA, USA) and were quantified by Qubit (Invitrogen, Waltham, MA, USA).

Validated libraries were pooled in equimolar quantities and sequenced as a paired-end 150-cycle run on an Illumina NextSeq2000. A total of 1548 million reads were generated, and raw reads were filtered for QV > 30 using an in-house phyton script. Filtered reads were aligned to unique clade-specific marker genes using MetaPhlAn 3 [80] to assess the taxonomic profile. The alignment was done indicating the closest name of species to the sequence (the best hit). The relative proportions calculated from MetaPhlAn were used to calculate relative abundances, alpha diversity measure (chao1 index) and beta diversity measures (Aitchison distance).

### 4.7. Statistical Analysis

#### 4.7.1. General Statistical Analysis

Statistical analysis was performed using the R software (version 4.0.2, R Core Team 2021), and different libraries, included in Bioconductor (version 3.11, Bioconductor project), were used. The continuous variables of biological assay were showed as mean ± standard error of the mean (SEM). After the normality study, parametric unpaired t-test was used for single statistical comparisons and repeated-measures analysis of variance (ANOVA) for multiple statistical comparisons repeated during time. In all the statistical comparisons, a two-tailed value of *p* < 0.05 was considered.

#### 4.7.2. Metabolomic Data Analysis

Individual comparisons between metabolites were determined by the MW test, because the variables follow the assumption of a non-parametric test. The p-value adjustment for multiple comparisons was carried out according to the BH correction considering a 5% false-discovery rate (FDR). The magnitude of difference between populations is presented as fold change (FC), which is relative to the control group. In parallel, a predictive analysis was done to evaluate the prediction power of the LPS-induced chronic inflammation model. On the one hand, PCA, an unsupervised multivariate data projection method, was performed to explore the native relationship between groups. On the other hand, OPLS-DA, a supervised multivariate data projection method, was calculated to explore the possible relationships between the observable variables (X) and the predicted variables or target (Y), extracting the maximum information reflecting the variation in the dataset. No data transformation was applied before conducting the analysis. The predictive performance of the test set was estimated by the Q2Y parameter calculated through cross-validation. The values of Q2 < 0 suggest a model with no predictive ability, 0 < Q2 < 0.5 suggests some predictive character and Q2 > 0.5 indicates good predictive ability [81]. The feature importance was calculated through the VIP, which reflects both the loading weights for each component and the variability of the response explained by the component. Additionally, the random forest classifier (RF) was calculated to sort the 10 most important metabolites that distinguish between the CON and LPS groups.

#### 4.7.3. Metagenomic Data Analysis

Centred log-ratio (CLR) normalization was performed before any statistical test. The beta diversity was calculated from the Aitchison distance, and PERMANOVA test was performed with 100 permutations to assess the differences between groups. The alpha diversity was calculated by Chao1 index. Taxonomic abundances were compared between experimental groups using the BH adjustment on MW test that is presented by relative abundance (%). The relative abundance was filtered to only include variables that were present above 0.01% in at least 3 samples [82]. The magnitude of difference between populations was determined by the determination of FC.

#### 4.7.4. Integration Data Analysis

Multiblock sPLS-DA is a holistic approach with the potential to find new biological insights not revealed by any single-data omics analysis, as some pathways are common to all data types, while other pathways may be specific to data. DIABLO implementation, which is built on the GCCA [83], in the mixOmics R package (version 6.18.1, mixOmics project), was used to integrate plasma and urine metabolome and microbiome [84]. The goal of the data integration with this method is to extract complementary information between omics datasets, resulting in an improved ability to associate biomarkers across multiple functional levels with the phenotype of interest. This statistical integrative framework facilitates the interpretation of complex analyses and provides significant biological insights.

To summarize, the first step is the parameter choice of the design matrix, the number of components and the number of variables to select: (1) a design matrix of 0.1 was used to focus primarily on the discrimination between the groups; (2) the perf function was used to estimate the performance of the model, and the overall error rates per component were displayed to select the optimal number of components; (3) the number of variables was chosen using the tune.block.splsda function that is run with 10-fold cross validation and repeated 10 times, and thus this tuning step led to a selection of 8 plasma metabolites, 6 urine metabolites, and 5 microbes. Thereafter, the final model was computed, and different sample and variable plots were performed. The circosPlot function represents the correlations between variables of different types, represented on the side quadrants that are built based on a similarity matrix, which was extended to the case of multiple data sets [85]. The resulting network from circus plot with a threshold above 0.7 was further analysed using the MEtScape [86] and NetworkAnalyzer [87] packages from Cytoscape (version 3.8.2, Institute of Systems Biology, Seattle, WA, USA) [88].

The final performance of the model was evaluated by the perf function using 10-fold cross-validation repeated 10 times. The ROC curve analysis was conducted to determine the optimal metabolite combination patterns that could correctly dichotomize the stressed and healthy groups at acceptable sensitivity and specificity (defined as greater than 80% for both). The AUC value was used as a measure of the prognostic accuracy. An AUC value of 1 indicates a perfect test due to absence of overlap of the test data between the control and anxiety state; an AUC value >0.85 was considered for inclusion in the model.

#### 4.7.5. Pathway Analysis

The resulting significant differential features were analysed through different databases to identify related pathways and elucidate the global effect on the metabolism of the LPS-induced inflammation model. The main database consulted was the Kyoto Encyclopaedia of Genes and Genomes (KEGG) [89]. To show those results, a mapping tool (version XMind 2020, XMind Ltd., Virginia, ON, Canada) was used to incorporate the information about pathway analysis.

## 5. Conclusions

In conclusion, intermittent (1- and 2-day intervals) and increasing (0.5, 5 and 7.5 mg/kg) dose IP administration of LPS thrice per week for 31 days induced chronic inflammation. In general, biometric and biochemical changes observed were in concordance to those seen in chronic inflammatory events. Thus, this model could be considered for the study of chronic inflammation in rodents, mimicking this risk for the development of inflammatory diseases.

The present study using omic approaches elucidates an altered profile associated with the current model of LPS-induced inflammation. In the metabolome, there is a clear disruption of the mitochondrial metabolism due to three key pathways in metabolism, which are β-oxidation of FAs, TCA cycle and 1C metabolism. Lipids related to β-oxidation play an important role in the unbalanced homeostasis during chronic inflammation. The main even SFA altered in our study was ChoE 18:0, which is associated with metabolic complications [38]. Other FAs related to pro-inflammatory activity such as ChoE 20:4 and anti-inflammatory activity were increased. In fact, the significant increase in ChoE 18:3 (precursor of ChoE 22:6) instead of ChoE 18:2 (precursor of ChoE 20:4) suggests that the synthesis of ChoE 22:6 reaction is favoured. Additionally, circulating phospholipids reported the long-term storage of FAs [43,44]. The decrease in TCA intermediaries (i.e., alpha-ketoglutarate, aconitic acid, malic acid, fumaric acid and succinic acid) was indicative of a systemic inhibition of the intracellular TCA cycle that could be associated with the regulation of immune response and activation of other energy pathways [58]. Additionally, the disruption of β-oxidation plays a key role in the TCA cycle due to the end product of this pathways being the starting point of TCA cycle [63]. Additionally, 1C metabolism is mainly represented in our model by glycine (circulating in plasma) and N,N-dimethylglycine (excreted in urine), which suggests a disruption in 1C metabolism [67].

Hence, the imbalance in FAs, TCA cycle intermediates and 1C intermediates metabolites are characteristics of our rodent model of chronic inflammation that leads to the disruption of homeostasis in mitochondrial metabolism (β-oxidation, TCA cycle and 1C metabolism). Those results provide novel clues on the impact of a model of chronic inflammation in the metabolism that tries to mimic chronic low-grade inflammation in humans.

## Figures and Tables

**Table 1:** Characteristics of the LPS-induced inflammation model. The results are presented as the mean ± SEM (*n* = 10 animals per group). The biometric parameters are represented as a ratio (g/kg BW × 100) to properly compare the parameters. The statistical comparisons among groups were conducted using *t*-student test, and fold change (FC) was calculated (LPS/CON). * *p* < 0.05 (significantly different) and ** *p* < 0.01 (high significantly different) compared with control. BW, body weight; RWAT, retroperitoneal white adipose tissue; MWAT, mesenteric white adipose tissue; MCP-1, monocyte chemoattractant protein-1; IL-6, interleukin-6; PGE2, prostaglandin E2; TNF-α, tumour necrosis factor alpha; TG, triglycerides; TC, total cholesterol; NEFAs, non-esterified fatty acids.

		Control	LPS	*p*-Value	FC
Biometric parameters	Initial BW (g)	303.37 ± 4.45	306.6 ± 3.13	0.67	1
	Final BW (g)	386.33 ± 10.28	365.29 ± 10.85	0.17	0.95
	Total food consumption (AUC)	604.09 ± 16.27	492.59 ± 17.04	<0.01 **	0.82
	RWAT/BW	1.83 ± 0.13	1.57 ± 0.15	0.21	0.86
	MWAT/BW	1.07 ± 0.09	0.96 ± 0.09	0.44	0.89
	Muscle/BW	0.63 ± 0.01	0.57 ± 0.02	0.02 *	0.90
	Liver/BW	2.73 ± 0.09	3.09 ± 0.07	<0.01 **	1.13
	Cecum/BW	1.22 ± 0.05	1.2 ± 0.04	0.73	0.98
Plasma parameters	MCP-1 (ng/mL)	4.59 ± 0.21	66.53 ± 19.14	<0.01 **	14.49
	IL-6 (ng/mL)	117.37 ± 5.97	172.80 ± 51.83	0.04 *	1.47
	PGE2 (ng/mL)	2.53 ± 2.67	4.42 ± 5.29	<0.01 **	1.75
	TNF-α (pg/mL)	8.75 ± 2.13	80.43 ± 23.68	0.03 *	9.18
	Glucose (mM)	101.09 ± 4.24	104.62 ± 2.27	0.47	1.03
	TG (mM)	107.76 ± 10.11	82.46 ± 4.15	0.03 *	0.76
	TC (mM)	63.02 ± 5.16	64.36 ± 3.31	0.83	1.02
	NEFAs (mM)	0.93 ± 0.08	0.77 ± 0.04	0.11	0.83
Liver biochemistry	Total lipids (mg/g)	34.53 ± 2.23	32.67 ± 1.99	0.54	0.95
	TC (mg/g)	1.32 ± 0.07	1.50 ± 0.14	0.26	1.14
	Phospholipids (mg/g)	11.53 ± 0.61	12.16 ± 0.91	0.57	1.05
	TG (mg/g)	3.39 ± 0.14	4.51 ± 0.47	0.04 *	1.33
Urine parameters	8-isoprostane (ng/mL)	0.81 ± 0.09	4.22 ± 0.70	<0.01 **	5.21

**Table 2:** Summary of the significant differential plasma metabolites in the LPS-induced inflammation model. CON and LPS groups (*n* = 10 animals per group) are represented by the relative abundances (AU). Relative abundances of metabolites are presented by the mean ± SEM. Plasma metabolites are sorted by *p*-value. The summary of the analysis is shown and includes the relative abundances of metabolites, *p*-value, *q*-value, VIP value, fold change (FC), the effect of the LPS vs. the CON group, and the related metabolic pathway. * *p* < 0.05 (significantly different) and ** *p* < 0.01 (highly significant difference) compared with control. Abbreviations: ChoE, cholesterol ester; LPC, lysophospholipid; PC, phosphatidylcholine; SM, sphingomyelin; TG, triglyceride; DG, diacylglycerol.

Metabolite	CON	LPS	*p*-Value	*q*-Value	VIP	RF	FC	Effect	Metabolic Pathway
Cholesterol	0.11 ± 0	0.15 ± 0.01	** <0.01	* 0.01	1.78	0	1.4	↑	Steroid biosynthesis
ChoE 18:0	0.09 ± 0.01	0.15 ± 0.01	** <0.01	* 0.01	1.69	0.04	1.7	↑	Fatty acids metabolism
ChoE 18:3	1.55 ± 0.12	2.48 ± 0.17	** <0.01	* 0.01	1.76	0.02	1.6	↑	
ChoE 20:4	59.73 ± 3.98	80.04 ± 3.21	** <0.01	* 0.03	1.76	0	1.3	↑	
ChoE 22:6	2.67 ± 0.19	4.28 ± 0.28	** <0.01	* 0.01	1.96	0.03	1.6	↑	
LPC 16:0 e	0.34 ± 0.03	0.52 ± 0.03	** <0.01	* 0.01	1.67	0	1.5	↑	Glycerophospholipid metabolism
LPC 18:0 e	0.07 ± 0	0.1 ± 0.01	** <0.01	* 0.01	1.61	0.03	1.4	↑	
PC 30:0	0.06 ± 0.01	0.09 ± 0.01	** <0.01	* 0.05	1.43	0.03	1.5	↑	
PC 32:0	0.7 ± 0.06	1.08 ± 0.05	** <0.01	* 0.01	1.67	0.02	1.5	↑	
PC 34:0	0.29 ± 0.02	0.46 ± 0.02	** <0.01	* 0.01	1.74	0.09	1.6	↑	
PC 34:1	4.84 ± 0.53	6.85 ± 0.44	* 0.01	* 0.03	1.36	0.04	1.4	↑	
PC 38:4	24.61 ± 1.61	35.21 ± 1.52	** <0.01	* 0.01	1.71	0.02	1.4	↑	
PC 40:4	0.25 ± 0.02	0.37 ± 0.04	** <0.01	* 0.02	1.47	0.02	1.5	↑	
SM 42:2	15.64 ± 1.49	23.35 ± 1.29	** <0.01	* 0.01	1.65	0	1.5	↑	Sphingolipid metabolism
SM 42:3	4.64 ± 0.39	7.07 ± 0.4	** <0.01	* 0.01	1.61	0.03	1.5	↑	
TG 54:7	5.21 ± 0.65	1.82 ± 0.39	** <0.01	* 0.01	1.56	0.08	0.3	↓	Lipid metabolism
DG 34:1	1.46 ± 0.08	1.84 ± 0.08	* 0.01	* 0.03	1.71	0.02	1.3	↑	
DG 36:4	3.42 ± 0.21	2.43 ± 0.11	** <0.01	* 0.02	1.89	0.02	0.7	↓	
α-ketoglutarate	2.05 ± 0.1	0.87 ± 0.09	** <0.01	* 0.01	2.05	0.06	0.4	↓	TCA cycle
Aconitic acid	0.02 ± 0	0.01 ± 0	** <0.01	* 0.02	1.64	0.04	0.5	↓	
Malic acid	0.44 ± 0.02	0.19 ± 0.02	** <0.01	* 0.01	2.02	0.08	0.4	↓	
Fumaric acid	0.63 ± 0.04	0.34 ± 0.04	** <0.01	* 0.01	1.95	0.02	0.5	↓	
Succinic acid	0.51 ± 0.02	0.41 ± 0.01	** <0.01	* 0.01	1.85	0.04	0.8	↓	
Glycine	4.57 ± 0.2	5.8 ± 0.36	* 0.03	* 0.01	1.55	0.03	1.3	↑	Glycine, serine and threonine metabolism

## Data Availability

The data presented in this study are available on request from the corresponding author. The data are not publicly available in the interest of performing more analysis for further publications together with more data.

## Supplementary Materials

The following supporting information can be downloaded at: https://www.mdpi.com/article/10.3390/ijms23052563/s1.
