# Supplementary material for: Imbalances in TCA, Short Fatty Acids and One-Carbon Metabolisms as Important Features of Homeostatic Disruption Evidenced by a Multi-Omics Integrative Approach of LPS-Induced Chronic Inflammation in Male Wistar Rats
Source: Int J Mol Sci. 2022 Feb 25;23(5):2563. doi: 10.3390/ijms23052563 (PMC8910732; doi:10.3390/ijms23052563)
Supplement: Supplementary file 1 [file ijms-23-02563-s001.zip › ijms-1541682-supplementary.pdf]

Supplementary Table S1. Statistical analysis of plasma metabolites in the LPS-induced inflammation model. CON and LPS groups (n = 10 animals per group) are represented by the relative abundances (AU). Relative abundances of metabolites are presented by the mean ± SEM. Plasma metabolites are sorted by p-value. The summary of analysis includes *p*-value, *q*-value, VIP value, random forest classifier (RF) and fold change (FC). The statistically significant p-values and q-values are highlighted in bold. Abbreviations: DG, diacylglycerol; ChoE, cholesterol ester; TG, triglyceride; PC, phosphatidylcholine; SM, sphingomyelin; LPC, lysophospholipid; PE, phosphatidylethanolamine.

Metabolite	CON	LPS	*p*-value	*q*-value	VIP	RF	FC
Alpha-ketoglutarate	2.05 ± 0.1	0.87 ± 0.09	**<0.01**	**0.01**	2.05	0.06	0.4
Malic acid	0.44 ± 0.02	0.19 ± 0.02	**<0.01**	**0.01**	2.02	0.08	0.4
ChoE (18:3)	1.55 ± 0.12	2.48 ± 0.17	**<0.01**	**0.01**	1.76	0.02	1.6
ChoE (22:6)	2.67 ± 0.19	4.28 ± 0.28	**<0.01**	**0.01**	1.96	0.03	1.6
LPC 16:0 e	0.34 ± 0.03	0.52 ± 0.03	**<0.01**	**0.01**	1.67	0.02	1.5
PC 34:0	0.29 ± 0.02	0.46 ± 0.02	**<0.01**	**0.01**	1.74	0.09	1.6
PC 32:0	0.7 ± 0.06	1.08 ± 0.05	**<0.01**	**0.01**	1.67	0.02	1.5
SM 42:3	4.64 ± 0.39	7.07 ± 0.4	**<0.01**	**0.01**	1.61	0.03	1.5
Fumaric acid	0.63 ± 0.04	0.34 ± 0.04	**<0.01**	**0.01**	1.95	0.02	0.5
LPC 18:0 e	0.07 ± 0	0.1 ± 0.01	**<0.01**	**0.01**	1.61	0.03	1.4
PC 38:4	24.61 ± 1.61	35.21 ± 1.52	**<0.01**	**0.01**	1.71	0.02	1.4
Succinic acid	0.51 ± 0.02	0.41 ± 0.01	**<0.01**	**0.01**	1.85	0.04	0.8
ChoE (18:0)	0.09 ± 0.01	0.15 ± 0.01	**<0.01**	**0.01**	1.69	0.04	1.7
TG 54:7	5.21 ± 0.65	1.82 ± 0.39	**<0.01**	**0.01**	1.56	0.08	0.3
Cholesterol	0.11 ± 0	0.15 ± 0.01	**<0.01**	**0.01**	1.78	-	1.4
SM 42:2	15.64 ± 1.49	23.35 ± 1.29	**<0.01**	**0.01**	1.65	-	1.5
DG 36:4	3.42 ± 0.21	2.43 ± 0.11	**<0.01**	**0.02**	1.89	0.02	0.7
Aconitic acid	0.02 ± 0	0.01 ± 0	**<0.01**	**0.02**	1.64	0.04	0.5
PC 40:4	0.25 ± 0.02	0.37 ± 0.04	**<0.01**	**0.02**	1.47	0.02	1.5
ChoE (20:4)	59.73 ± 3.98	80.04 ± 3.21	**<0.01**	**0.03**	1.76	-	1.3
Glycine	4.57 ± 0.2	5.8 ± 0.36	**<0.01**	**0.03**	1.55	0.03	1.3
DG 34:1	1.46 ± 0.08	1.84 ± 0.08	**0.01**	**0.03**	1.71	0.02	1.3
PC 34:1	4.84 ± 0.53	6.85 ± 0.44	**0.01**	**0.03**	1.36	0.04	1.4
PC 30:0	0.06 ± 0.01	0.09 ± 0.01	**0.01**	**<0.05**	1.43	0.03	1.5
SM 34:1	17.9 ± 1.47	23.29 ± 0.87	**0.01**	0.06	1.36	0.03	1.3
Hydroxyproline	1.03 ± 0.03	0.81 ± 0.06	**0.01**	0.07	1.30	0.01	0.8
Alanine	0.78 ± 0.05	1.12 ± 0.13	**0.02**	0.08	1.43	-	1.4
ChoE (16:0)	0.94 ± 0.07	1.22 ± 0.06	**0.02**	0.08	1.40	-	1.3
TG 52:5	8.63 ± 1.04	5.14 ± 0.63	**0.02**	0.09	1.27	0.02	0.6
PC 36:2	12.78 ± 1.21	16.44 ± 1.07	**0.03**	0.13	1.11	-	1.3
Pyruvic acid	26.89 ± 2.5	40.27 ± 5.19	**0.03**	0.13	0.87	0.01	1.5
Glutamine	0.04 ± 0	0.02 ± 0.01	**0.04**	0.14	1.31	-	0.5
TG 52:6	1.16 ± 0.15	0.74 ± 0.09	**0.04**	0.14	1.03	-	0.6
Threonine	2.15 ± 0.07	2.69 ± 0.22	**0.04**	0.14	1.29	-	1.3
Aspartic acid	0.68 ± 0.04	0.82 ± 0.05	**<0.05**	0.14	1.10	-	1.2
Histidine	0.07 ± 0.01	0.1 ± 0.01	**<0.05**	0.14	0.70	-	1.4
Leucine	0.15 ± 0.01	0.12 ± 0.01	**<0.05**	0.14	0.94	-	0.8
PC 32:2	0.24 ± 0.03	0.33 ± 0.03	**<0.05**	0.14	1.09	0.02	1.4

PC 36:4	23.43 ± 1.55	28.01 ± 1.21	**<0.05**	0.14	1.05	0.01	1.2
SM 35:1	0.18 ± 0.02	0.23 ± 0.02	**<0.05**	0.14	1.06	-	1.3
TG 50:4	1.82 ± 0.28	1.02 ± 0.15	0.05	0.16	1.05	-	0.6
TG 54:4	14.62 ± 1.88	9.97 ± 1.44	0.05	0.16	0.93	-	0.7
LPC 18:0	40.88 ± 2.53	51.91 ± 3.4	0.06	0.19	1.19	0.01	1.3
PC 40:5	0.62 ± 0.06	0.77 ± 0.03	0.06	0.19	1.09	-	1.2
ChoE (18:2)	19.3 ± 1.52	22.57 ± 0.9	0.08	0.21	0.99	0.02	1.2
Tryptophan	0.82 ± 0.16	1.32 ± 0.18	0.08	0.21	0.91	-	1.6
ChoE (22:5)	0.94 ± 0.09	1.14 ± 0.06	0.09	0.24	1.16	-	1.2
Glucose	0.22 ± 0.01	0.24 ± 0.01	0.10	0.25	0.72	-	1.1
PC 33:0	0.04 ± 0	0.04 ± 0	0.10	0.25	0.94	-	1
PC 38:3	0.96 ± 0.12	1.14 ± 0.1	0.10	0.25	0.85	-	1.2
Proline	0.65 ± 0.04	0.57 ± 0.03	0.10	0.25	0.76	-	0.9
SM 38:1	0.44 ± 0.04	0.55 ± 0.04	0.10	0.25	1.05	-	1.3
TG 52:3	60.57 ± 6.94	43.35 ± 4.91	0.10	0.25	0.93	-	0.7
2-hydroxyglutaric	0.8 ± 0.07	0.62 ± 0.08	0.12	0.27	1.03	-	0.8
Glycolic acid	12.61 ± 0.46	11.65 ± 0.43	0.12	0.27	0.73	-	0.9
PC 31:0	0.04 ± 0	0.05 ± 0	0.12	0.27	0.84	-	1.3
SM 40:1	4.31 ± 0.35	5.17 ± 0.34	0.12	0.27	0.96	0.01	1.2
TG 48:1	1.37 ± 0.32	2.69 ± 0.77	0.12	0.27	1.00	-	2
Fructose	1.85 ± 0.16	1.54 ± 0.11	0.14	0.29	0.78	-	0.8
PC 32:1	0.77 ± 0.13	1.13 ± 0.17	0.14	0.29	1.00	-	1.5
SM 32:1	0.2 ± 0.02	0.22 ± 0.01	0.14	0.29	0.63	-	1.1
Threonic acid	1.71 ± 0.07	1.52 ± 0.1	0.14	0.29	0.98	-	0.9
LPC 18:1	13.7 ± 1.28	11.38 ± 0.55	0.16	0.33	0.74	-	0.8
DG 34:2	2.44 ± 0.08	2.51 ± 0.07	0.19	0.37	0.49	-	1
TG 54:3	6.14 ± 0.73	4.92 ± 0.59	0.19	0.37	0.55	-	0.8
PC 33:1	0.1 ± 0.01	0.12 ± 0.01	0.21	0.40	0.77	-	1.2
TG 50:1	3.92 ± 0.52	6.87 ± 1.63	0.21	0.40	1.02	-	1.8
Urea	0.92 ± 0.03	1.01 ± 0.05	0.21	0.40	0.79	-	1.1
Phenylalanine	2.08 ± 0.06	2.24 ± 0.09	0.24	0.44	0.99	0.01	1.1
SM 36:1	1.16 ± 0.09	1.41 ± 0.11	0.24	0.44	0.92	-	1.2
Beta-alanine	0.07 ± 0.01	0.09 ± 0.02	0.27	0.49	0.70	0.01	1.3
Oleic acid	1.61 ± 0.05	1.75 ± 0.09	0.27	0.49	0.86	-	1.1
Methionine	0.12 ± 0.01	0.13 ± 0.01	0.31	0.52	0.58	-	1.1
Ornithine	2.55 ± 0.24	2.78 ± 0.25	0.31	0.52	0.37	-	1.1
Tyrosine	0.26 ± 0.05	0.33 ± 0.06	0.31	0.52	0.72	-	1.3
Lactic acid	5.28 ± 0.22	4.95 ± 0.21	0.34	0.57	0.56	-	0.9
LPC 18:2	40.43 ± 4.01	34.65 ± 2.35	0.34	0.57	0.58	-	0.9
SM 39:1	0.18 ± 0.02	0.19 ± 0.02	0.34	0.57	0.46	-	1.1
Isoleucine	0.43 ± 0.03	0.59 ± 0.23	0.38	0.62	0.07	-	1.4
SM 34:2	1.48 ± 0.13	1.63 ± 0.07	0.38	0.62	0.66	-	1.1
Glutamic acid	0.09 ± 0.01	0.11 ± 0.01	0.43	0.65	0.71	0.01	1.2
LPC 15:0	0.74 ± 0.08	0.67 ± 0.05	0.43	0.65	0.37	-	0.9
TG 46:1	0.11 ± 0.03	0.16 ± 0.05	0.43	0.65	0.58	-	1.5

TG 48:2	1.13 ± 0.28	1.45 ± 0.37	0.43	0.65	0.48	-	1.3
PC 35:2	0.39 ± 0.04	0.44 ± 0.04	0.47	0.70	0.44	-	1.1
SM 42:1	17.87 ± 1.52	19.93 ± 1.01	0.47	0.70	0.70	-	1.1
TG 50:2	14.09 ± 2.18	18.19 ± 3.48	0.47	0.70	0.61	-	1.3
ChoE (17:1)	0.02 ± 0.01	0.02 ± 0.01	0.52	0.75	0.23	-	1
TG 52:1	0.96 ± 0.1	1.19 ± 0.21	0.52	0.75	0.61	-	1.2
Alpha-tocopherol	0.42 ± 0.04	0.48 ± 0.06	0.57	0.77	0.43	-	1.1
Asparagine	0.18 ± 0.03	0.16 ± 0.02	0.57	0.77	0.50	-	0.9
ChoE (22:4)	9.95 ± 0.84	11.14 ± 0.74	0.57	0.77	0.69	-	1.1
Glucose-6-phosphate	0.67 ± 0.08	0.82 ± 0.15	0.57	0.77	0.29	-	1.2
TG 46:0	0.1 ± 0.01	0.15 ± 0.04	0.57	0.77	0.65	-	1.5
Valine	1.25 ± 0.05	1.38 ± 0.13	0.57	0.77	0.61	-	1.1
ChoE (18:1)	2.65 ± 0.23	2.51 ± 0.14	0.62	0.82	0.20	-	0.9
DG 36:2	1.63 ± 0.07	1.66 ± 0.07	0.62	0.82	0.44	-	1
ChoE (20:2)	1.81 ± 0.19	1.67 ± 0.16	0.68	0.84	0.30	-	0.9
Lysine	0.11 ± 0.03	0.12 ± 0.03	0.68	0.84	0.44	-	1.1
SM 33:1	0.34 ± 0.03	0.37 ± 0.01	0.68	0.84	0.63	-	1.1
TG 48:0	0.67 ± 0.08	0.92 ± 0.22	0.68	0.84	0.62	-	1.4
TG 50:0	0.17 ± 0.05	0.22 ± 0.06	0.68	0.84	0.42	-	1.3
TG 50:3	8.63 ± 1.48	7.69 ± 1.39	0.68	0.84	0.14	-	0.9
Glyceric acid	0.92 ± 0.06	0.98 ± 0.1	0.73	0.89	0.13	-	1.1
TG 46:2	0.06 ± 0.02	0.07 ± 0.02	0.73	0.89	0.28	-	1.2
TG 48:3	0.32 ± 0.07	0.28 ± 0.06	0.73	0.89	0.07	-	0.9
3-hydroxybutiric acid	2.04 ± 0.13	2.08 ± 0.19	0.79	0.92	0.01	-	1
ChoE (17:0)	0.02 ± 0.01	0.02 ± 0.01	0.79	0.92	0.27	-	1
LPC 20:0	0.26 ± 0.02	0.27 ± 0.01	0.79	0.92	0.16	-	1
TG 54:2	3.67 ± 0.47	3.45 ± 0.45	0.79	0.92	0.01	-	0.9
PC 38:2	0.2 ± 0.02	0.21 ± 0.01	0.85	0.95	0.41	-	1.1
Serine	0.5 ± 0.03	0.56 ± 0.08	0.85	0.95	0.25	-	1.1
TG 51:2	0.86 ± 0.13	0.78 ± 0.12	0.85	0.95	0.09	-	0.9
TG 52:2	19.11 ± 2.93	18.4 ± 2.94	0.85	0.95	0.08	-	1
TG 54:6	21.68 ± 2.17	20.48 ± 1.86	0.85	0.95	0.24	-	0.9
Fructose-6-phosphate	0.46 ± 0.06	0.54 ± 0.12	0.91	0.98	0.01	-	1.2
Ribose	26.67 ± 3.59	26.14 ± 3.57	0.91	0.98	0.05	-	1
SM 41:1	4.85 ± 0.35	5.1 ± 0.3	0.91	0.98	0.39	0.01	1.1
SM 41:2	1.18 ± 0.11	1.19 ± 0.05	0.91	0.98	0.19	-	1
Citric acid	3.93 ± 0.12	3.94 ± 0.24	0.97	1.00	0.02	-	1
Glycerol	3.6 ± 0.28	3.56 ± 0.26	0.97	1.00	0.05	-	1
LPC 16:0	73.27 ± 5.3	75 ± 3.44	0.97	1.00	0.19	-	1
SM 36:2	0.42 ± 0.04	0.43 ± 0.02	0.97	1.00	0.24	-	1
ChoE (16:1)	0.59 ± 0.09	0.58 ± 0.08	1.00	1.00	0.13	-	1
DG 34:3	0.73 ± 0.06	0.7 ± 0.05	1.00	1.00	0.11	-	1
LPC 16:1 e	0.11 ± 0.01	0.12 ± 0.01	1.00	1.00	0.43	-	1.1
SM 40:2	0.77 ± 0.07	0.78 ± 0.04	1.00	1.00	0.18	-	1
SM 43:1	1.59 ± 0.14	1.61 ± 0.07	1.00	1.00	0.20	0.01	1

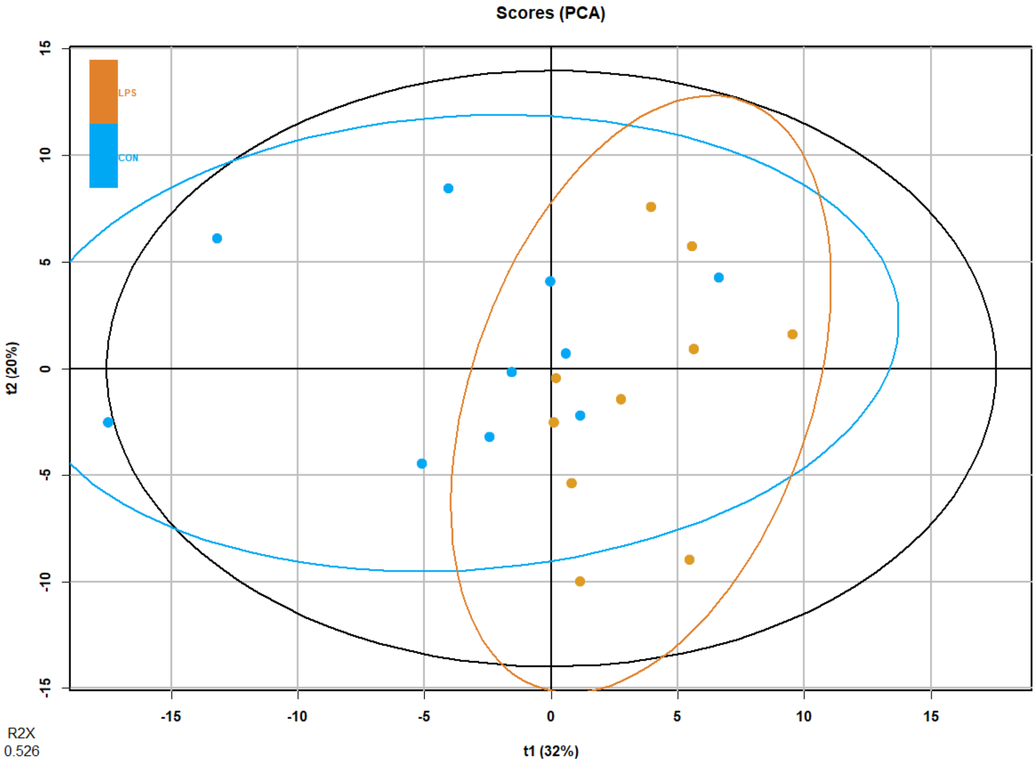

Supplementary Figure S1. PCA of plasma metabolomics in the LPS-induced inflammation model. Blue represents CON group and orange LPS group (n = 10 animals per group).

Supplementary Table S2. Statistical analysis of urine metabolites in the LPS-induced inflammation model. CON and LPS groups (n = 10 animals per group) are represented by the relative abundances (AU). Relative abundances of metabolites are presented by the mean ± SEM. Plasma metabolites are sorted by p-value. The summary of univariant and multivariate analysis is shown including p-value, q-value, random forest classifier (RF) and fold change (FC) The statistically significant *p*-values and *q*-values (< 0.05) are highlighted in bold. Abbreviations: 3-HPPA, 3-hydroxyphenylpropionate.

Metabolite	CON	LPS	*p*-value	*q*-value	RF	FC
N,N-Dimethylglycine	3.59 ± 0.41	7.47 ± 1.44	**<0.01**	0.25	0.17	2.1
Dimethylsulfone	14.66 ± 1.11	11.93 ± 0.67	0.07	0.78	0.09	0.8
Fucose	2.64 ± 1.44	2.02 ± 0.33	0.13	0.78	0.06	0.8
Fumarate	3.87 ± 0.35	5.08 ± 0.82	0.13	0.78	0.06	1.3
Citrate	291.02 ± 26.18	234.34 ± 20.97	0.18	0.78	0.05	0.8
Formate	2.77 ± 0.22	3.72 ± 0.55	0.21	0.78	0.04	1.3
Succinate	31.64 ± 2.28	35.55 ± 2.06	0.24	0.78	0.04	1.1
Choline	6.41 ± 0.8	7.9 ± 0.76	0.31	0.78	0.04	1.2
Hippurate	79.56 ± 5.07	72.33 ± 5.2	0.31	0.78	0.04	0.9
Isoleucine	14.69 ± 0.78	16.18 ± 1.45	0.31	0.78	0.04	1.1
N-Acetylglycoproteins	102.1 ± 4.33	106.23 ± 5.52	0.31	0.78	0.03	1
Glucose	3.23 ± 0.4	2.94 ± 0.17	0.35	0.78	0.03	0.9
3-HPPA Sulfate	4.01 ± 0.7	5.83 ± 1.28	0.39	0.78	0.03	1.5

4-Guanidinobutanoate	12.38 ± 0.66	13.04 ± 0.47	0.39	0.78	0.03	1.1
Lactate	9.66 ± 1.16	9.76 ± 1.61	0.39	0.78	0.03	1
Methylamine	4.32 ± 0.7	3.69 ± 0.38	0.39	0.78	0.02	0.9
Trigonelline	1.26 ± 0.07	1.15 ± 0.06	0.44	0.82	0.02	0.9
2-Oxoglutarate	373.05 ± 42.57	341.04 ± 34.71	0.49	0.82	0.02	0.9
Alanine	5.44 ± 0.57	6.03 ± 0.41	0.54	0.82	0.02	1.1
Allantoin	213.8 ± 14.49	229.6 ± 9.79	0.54	0.82	0.02	1.1
Pseudouridine	9.32 ± 0.79	10.07 ± 0.51	0.54	0.82	0.02	1.1
Glyoxylic acid	0.33 ± 0.04	0.33 ± 0.04	0.60	0.87	0.02	1
Acetate	5.55 ± 0.89	6.43 ± 1.72	0.65	0.87	0.01	1.2
Phenylacetylglycine	55.78 ± 6.22	58.17 ± 5.19	0.65	0.87	0.01	1
3-HPPA	14.37 ± 1.45	14.22 ± 1.52	0.78	0.89	0.01	1
Creatinine	347.25 ± 13.61	346.44 ± 17.76	0.78	0.89	0.01	1
1-Methylnicotinamide	0.12 ± 0.04	0.14 ± 0.04	0.84	0.89	0.01	1.2
Indoxyl sulfate	5.51 ± 0.46	5.59 ± 0.56	0.84	0.89	0.01	1
Pyruvate	4.81 ± 0.48	5.37 ± 0.82	0.84	0.89	0.01	1.1
Taurine	389.61 ± 66.45	372.46 ± 40.77	0.84	0.89	0.01	1
Glycine	11.22 ± 1.78	11.04 ± 1.57	0.90	0.93	-	1
Betaine	16.7 ± 2.4	16.23 ± 1.3	0.97	0.99	-	1

Supplementary Figure S2. PCA of urine metabolomics in the LPS-induced inflammation model. Blue represents CON group and orange LPS group (n = 10 animals per group).

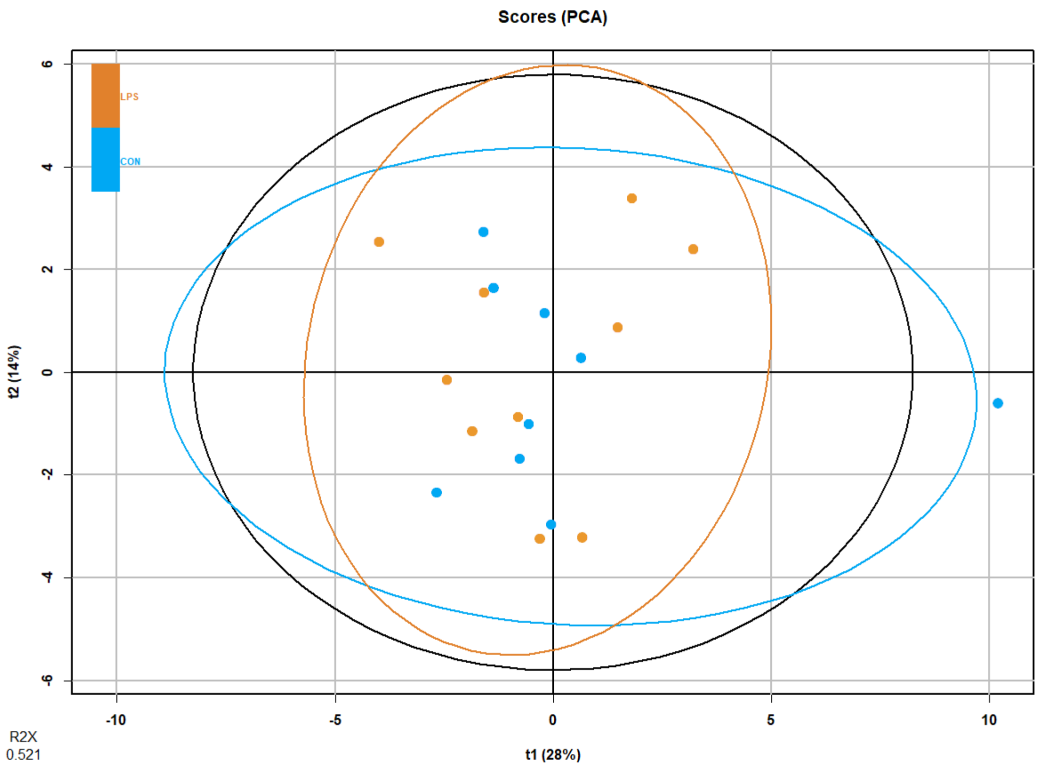

Supplementary Table S3. Summary of bacteria species in the LPS-induced inflammation model. The summary of univariant analysis of CON group and LPS group (n = 8 animals per group) includes the results of MW test (*p*-value), MW corrected by BH (*q*-value) and FC. The alignment was done indicating the closest name of specie to the sequence (the best hit). Taxonomic data is presented by the mean of relative abundance (%) and shorted by p-value. The statistically significant values (< 0.05) are highlighted in bold.

Specie	CON	LPS	*p*-value	*q*-value	FC
Muribaculum intestinale	21.46%	28.03%	**0.03**	0.55	1.3
Lachnospiraceae bacterium A4	0.13%	0.32%	**0.03**	0.55	2.6
Firmicutes bacterium ASF500	2.27%	0.36%	**0.03**	0.61	0.2
Akkermansia muciniphila	14.42%	17.86%	0.05	0.76	1.2
Muribaculaceae bacterium DSM 103720	18.50%	27.66%	0.06	0.82	1.5
Bacteroides uniformis	4.94%	6.21%	0.09	0.82	1.3
Lachnospiraceae bacterium COE1	1.06%	0.09%	0.11	0.82	0.1
Anaerotruncus sp G3 2012	5.25%	2.99%	0.16	0.90	0.6
Romboutsia ilealis	0.14%	0.25%	0.16	0.90	1.8
Oscillibacter sp 1 3	1.66%	0.68%	0.25	0.96	0.4
Bifidobacterium pseudolongum	0.54%	<0.01%	0.25	0.96	-
Lachnospiraceae bacterium 10 1	0.08%	<0.01%	0.25	0.96	-
Acutalibacter muris	0.03%	<0.01%	0.25	0.96	-
Escherichia coli	12.23%	4.16%	0.28	0.96	0.3
Parabacteroides goldsteinii	4.27%	2.33%	0.32	0.96	0.5
Bacteroides caecimuris	0.11%	0.27%	0.32	0.96	2.5
Mucispirillum schaedleri	12.74%	4.89%	0.36	0.96	0.4
Lactobacillus murinus	0.14%	3.90%	0.48	0.96	27.2
Enterorhabdus caecimuris	0.02%	<0.01%	0.48	0.96	-

Supplementary Table S4. Summary of virus species in the LPS-induced inflammation model. The summary of univariant analysis of CON group and LPS group (n = 8 animals per group) includes the results of MW test (*p*-value), MW corrected by BH (*q*-value) and FC. The alignment was done indicating the closest name of specie to the sequence (the best hit). Taxonomic data is presented by the mean of relative abundance (%) and shorted by p-value. The statistically significant values (< 0.05) are highlighted in bold.

Genera	Specie	CON	LPS	p-value	q-value	FC
Alphabaculovirus	*Chrysodeixis chalcites nucleopolyhedrovirus*	1.00%	0.27%	0.02	0.38	0.3
Siphoviridae unclassified	*Lactobacillus prophage Lj928*	1.73%	0.00%	0.11	0.92	-
Gammaretrovirus	*Abelson murine leukemia virus*	8.95%	13.57%	0.16	0.97	1.5
Gammaretrovirus	*Murine osteosarcoma virus*	15.00%	20.02%	0.16	0.97	1.3
Simplexvirus	*Human alphaherpesvirus 2*	0.14%	<0.01%	0.22	0.99	-
Myoviridae unclassified	*Lactobacillus prophage Lj771*	13.61%	10.29%	0.28	1.00	0.8
Cyprinivirus	*Anguillid herpesvirus 1*	5.18%	3.38%	0.28	1.00	0.7
Cyprinivirus	*Cyprinid herpesvirus 3*	41.97%	38.53%	0.32	1.00	0.9
Rhadinovirus	*Alcelaphine gammaherpesvirus 1*	4.74%	6.10%	0.28	1.00	1.3
Herpesviridae unclassified	*Ateline gammaherpesvirus 3*	6.79%	7.04%	0.32	1.00	1.0
Gammaretrovirus	*Koala retrovirus*	0.34%	0.40%	0.40	1.00	1.2
Potyvirus	*Zantedeschia mild mosaic virus*	<0.01%	0.06%	0.32	1.00	-
Varicellovirus	*Bovine alphaherpesvirus 1*	0.56%	0.10%	0.48	1.00	0.2

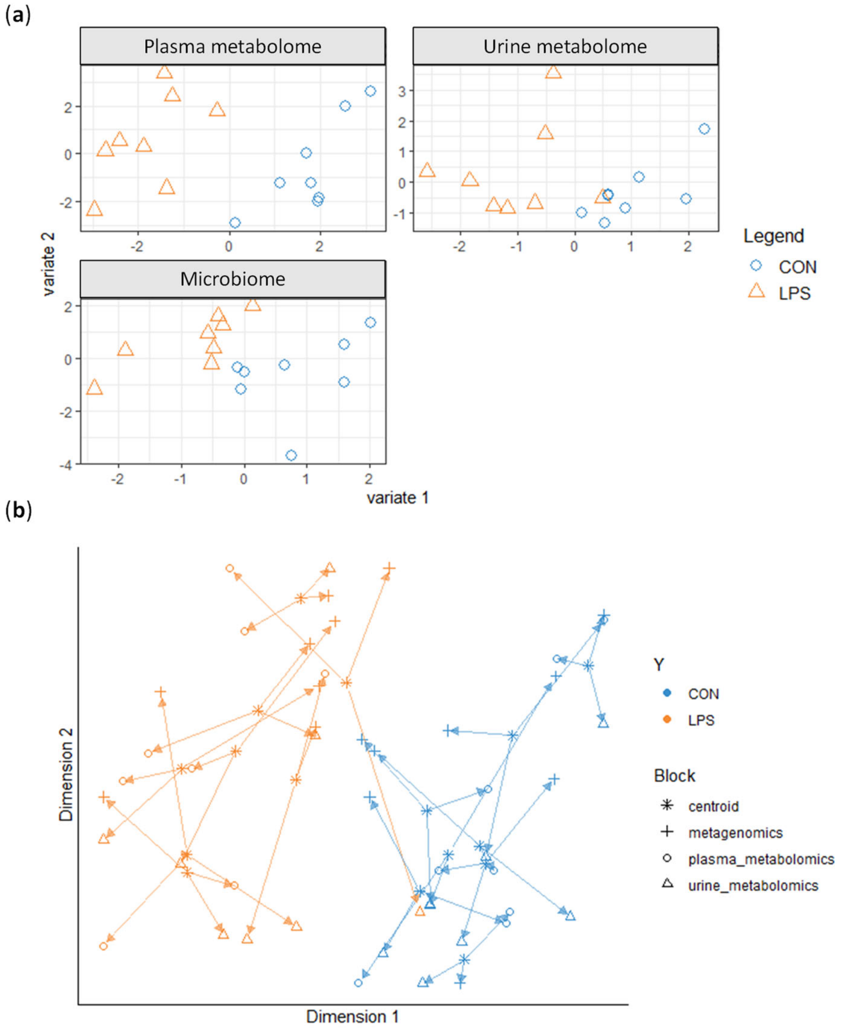

Supplementary Figure S3. Integration of plasma metabolomics, urine metabolomics and metagenomics data using DIABLO in the LPS-induced inflammation model. (a) The sample plot projects each sample into the space spanned by the components of each block. The first components from each data set are highly correlated to each other. (b) Arrow plot: the start of the arrow indicates the centroid between all data sets for a given sample and the tips of the arrows the location of that sample in each omic, highlighting the agreement between the 3 data sets at the sample level. LPS in orange and CON in blue.

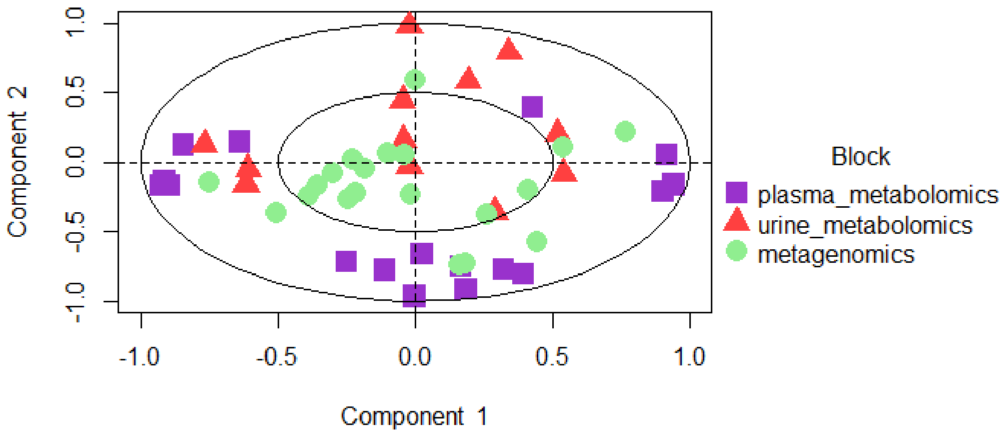

Supplementary Figure S4. Correlation circle plot in the LPS-induced inflammation model (DIABLO). This plot highlights the contribution of each selected variable to component 1 and 2. Clusters of points indicate a strong correlation between variables. Each colour and shape indicate the type of features: i.e., plasma metabolites (purple square), urine metabolites (red triangle) and finally, bacteria and virus species (green circle).

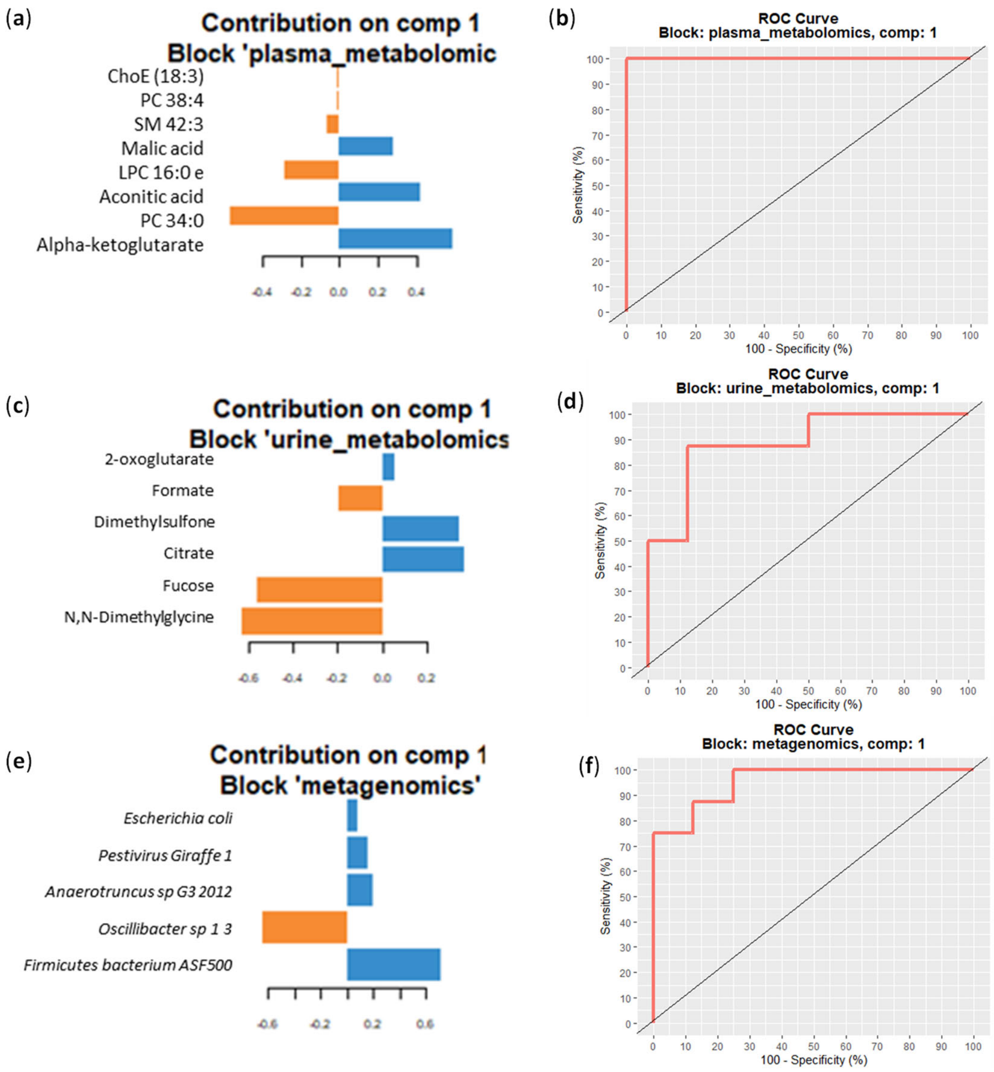

Supplementary Figure S5. Feature integration in plasma metabolomics, urine metabolomics and metagenomics in the LPS-induced inflammation model (DIABLO). (a) (c) (e) Feature impact in each data set in component 1. (b) (d) (f) ROC curve and AUC averaged using one-vs-all comparisons in the different data set. LPS in orange and CON in blue.
